# How To Make Nitroaromatic Compounds Glow: Next‐Generation Large X‐Shaped, Centrosymmetric Diketopyrrolopyrroles

**DOI:** 10.1002/anie.202005244

**Published:** 2020-07-10

**Authors:** Kamil Skonieczny, Ilias Papadopoulos, Dominik Thiel, Krzysztof Gutkowski, Philipp Haines, Patrick M. McCosker, Adèle D. Laurent, Paul A. Keller, Timothy Clark, Denis Jacquemin, Dirk M. Guldi, Daniel T. Gryko

**Affiliations:** ^1^ Institute of Organic Chemistry PAS. 44/52 Kasprzaka 01-224 Warsaw Poland; ^2^ Department of Chemistry and Pharmacy & Interdisciplinary Center for Molecular Materials (ICMM) Friedrich-Alexander-Universität Erlangen-Nürnberg (FAU) Egerlandstrasse 3 91058 Erlangen Germany; ^3^ Department of Chemistry and Pharmacy & Computer-Chemie-Center (CCC) Friedrich-Alexander-Universität Erlangen-Nürnberg Nägelsbachstrasse 25 91052 Erlangen Germany; ^4^ School of Chemistry & Molecular Bioscience, Molecular Horizons University of Wollongong Wollongong NSW 2522 Australia; ^5^ Illawarra Health & Medical Research Institute Wollongong NSW 2522 Australia; ^6^ Université de Nantes CNRS CEISAM UMR 6230 Nantes France

**Keywords:** diketopyrrolopyrroles, donor–acceptor systems, dyes/pigments, fluorescence, lactams

## Abstract

Red‐emissive π‐expanded diketopyrrolopyrroles (DPPs) with fluorescence reaching λ=750 nm can be easily synthesized by a three‐step strategy involving the preparation of diketopyrrolopyrrole followed by N‐arylation and subsequent intramolecular palladium‐catalyzed direct arylation. Comprehensive spectroscopic assays combined with first‐principles calculations corroborated that both N‐arylated and fused DPPs reach a locally excited (S_1_) state after excitation, followed by internal conversion to states with solvent and structural relaxation, before eventually undergoing intersystem crossing. Only the structurally relaxed state is fluorescent, with lifetimes in the range of several nanoseconds and tens of picoseconds in nonpolar and polar solvents, respectively. The lifetimes correlate with the fluorescence quantum yields, which range from 6 % to 88 % in nonpolar solvents and from 0.4 % and 3.2 % in polar solvents. A very inefficient (T_1_) population is responsible for fluorescence quantum yields as high as 88 % for the fully fused DPP in polar solvents.

## Graphical abstract

**Go with the glow**: A two‐step process has been developed that transforms parent diketopyrrolopyrrole into structures with up to 10 fused rings. The absorptions are bathochromically shifted to the deep‐red region and strong red emission is seen, even in nonpolar solvents, despite the presence of NO_2_ groups.

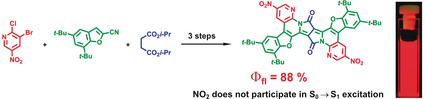

## Introduction

A significant number of heterocyclic analogues of nanographenes are known to date,[^1^, ^2^] but the overwhelming majority lack electron‐withdrawing and/or electron‐donating groups and remain unpolarized. This can be attributed to significant difficulties originating from the lack of compatibility between the required functional groups and the synthetic methods used.

To achieve such a goal, rather than decorating large π‐systems with electron‐withdrawing and electron‐donating substituents, one may envision the use of highly polarized heterocyclic scaffolds. In this regard, a family of cross‐conjugated, donor–acceptor chromophores is plausibly the best candidate. Among the cross‐conjugated chromophores,^[3]^ such as indigos,^[4]^ isoindigos,^[5]^ bay‐annulated indigos,^[6]^ quinacridones,^[4a]^ epindolindiones,[^4b^, ^6^] and dipyrrolonaphthyridinediones,^[7]^ diketopyrrolopyrroles (DPPs)[^8^, ^9^, ^10^] are unique for two major reasons: 1) they are the most intensively probed material in the field of organic electronics and photonics in recent decades,[^11^, ^12^] and 2) its synthetic chemistry is the most developed, thereby opening, in principle, unlimited possibilities for tailored modifications.^[13]^

**Figure 1:**
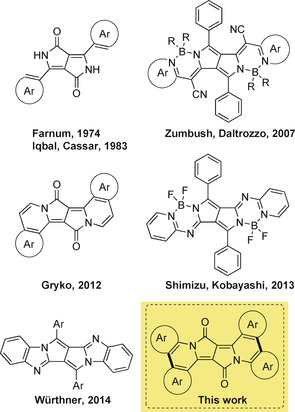
The history of π‐expanded diketopyrrolopyrroles.

The functional groups present in the core structure of DPPs serve as a starting point towards the design of novel architectures possessing π‐expanded chromophores.^[14]^ Only four different strategies have been presented so far that aim at the π‐expansion of DPPs .[^15^, ^16^, ^17^, ^18^] In each case, novel materials with intriguing photophysical features were obtained (Figure 1).^[19]^ It is clear that new combinations and fusions of DPPs with other scaffolds may provide access to unprecedented functional dyes, which may retain some of the advantageous properties of DPP, but extend their typical photophysics. Along these lines, we envisioned another strategy that would enable unlimited structural modifications and would lead, in turn, to unprecedented photophysical properties. In parallel, and in light of nitro functional groups acting as a notorious, although quite mysterious, quencher of fluorescence,^[20]^ we were interested in shedding light onto the influence of nitro groups on the emission of DPPs. Last, but not least, we were eager to probe the effect of symmetry. This last interest relates to the specific framework of symmetry breaking in quadrupolar centrosymmetric dyes.^[21]^

Herein, we provide the first examples of X‐shaped, highly polarized, π‐expanded diketopyrrolopyrroles synthesized by the *N*‐arylation of DPPs followed by an intramolecular direct arylation of the aromatic rings that are present in these building blocks.

## Results and Discussion

### Design and Synthesis

The construction of π‐expanded DPPs requires the installation of two aryl substituents possessing bromine atoms adjacent to the linking positions on the DPP nitrogen atoms. This calls for 1‐fluoro‐2‐bromobenzenes. One of the key problems in the chemistry of DPPs is, however, the limited scope of *N*‐arylation. The ubiquitous presence of bis‐*N*‐alkylated DPPs[^8^, ^10^, ^22^] contrasts with the scarcity of bis‐*N*‐arylated DPPs.[^23^, ^24^, ^25^]

The most intuitive approach is based on the reaction between DPP and the corresponding aryl fluoride in the presence of a base. Thus, to accomplish our goal, we began by testing the activity of commercially available 3‐bromo‐4‐fluoronitrobenzene for the arylation of DPP. In this case, however, we only obtained a monoarylation product in a low yield (15 %).

In light of this result, we resorted to the 2‐fluoropyridine derivative, which would be more reactive in an S_N_Ar reaction. 3‐Bromo‐2‐fluoro‐5‐nitropyridine was generated in situ by heating 3‐bromo‐2‐chloro‐5‐nitropyridine (**1**) in DMF in the presence of KF for two hours.^[26]^

**Scheme 1:**
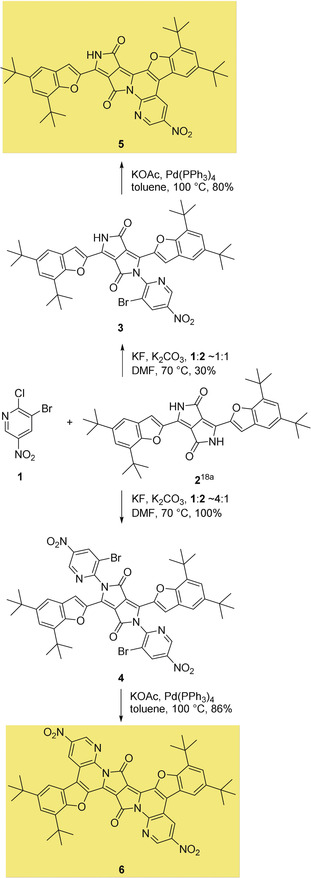
Synthesis of π‐expanded diketopyrrolopyrroles **5** and **6**.

It turned out that DPP **2** reacted easily with 3‐bromo‐2‐fluoro‐5‐nitropyridine in the presence of potassium carbonate in DMF at 70 °C to give the corresponding *N*‐arylated DPPs, which enabled the implementation of a “one‐pot” procedure (Scheme 1). The reaction with 1.2 equivalents (or less) of the aryl fluoride produced a mixture of products which, in addition to the expected monoarylated DPP **3**, also contained unreacted substrate and a significant amount of diarylated DPP **4**. By increasing the amount of aryl fluoride to 4 equivalents, we obtained bis‐*N*,*N*‐arylated DPP **4** in a pure form by crystallization from the reaction mixture. The bisarylation reaction was essentially quantitative in this case. In the next step, both **3** and **4** were subjected to the KOAc and Pd(PPh_3_)_4_ conditions developed by various groups for the intramolecular, direct arylation of aromatic and heteroaromatic systems.^[27]^ We obtained both π‐expanded products **5** and **6** with good yields when the reaction was carried out for 24 h in dry toluene at 100 °C.

### Steady‐State Absorption Spectroscopy

**Figure 2:**
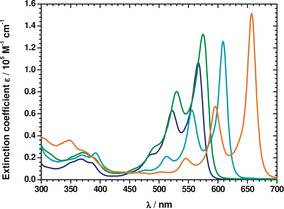
Room‐temperature absorption spectra of **3** (dark blue), **4** (green), **5** (pale blue), and **6** (orange) in toluene.

Absorption spectroscopy with dyes **3**–**6** in toluene reveal S_0_‐S_1_ absorption maxima in the λ=450 to 660 nm range (Table 1, Figure 2). The size of the π‐conjugated system affects the shifts of the absorption maxima to longer wavelengths, with the following order observed: **3**<**4**<**5**<**6**. DPP **3** has the smallest π‐conjugated system and gives rise to absorption maxima at λ=453, 489, 522, and 567 nm as a result of vibronic couplings (see below).

**Table 1:** Fundamental photophysical properties of DPPs **3**–**6** in toluene.

Compound	λ_abs_ [nm]	*ϵ* (10^4^ m ^−1^ cm^−1^)	λ_em_ [nm]	Φ_fl_ [%]
**3**	567, 522	10.8	575, 625	6
**4**	575, 530	13.0	585, 636	35
**5**	609, 556	12.6	615, 676	39
**6**	657, 595	14.6	663, 735	88

Di‐*N*‐arylated DPP **4** has the lowest energy absorption maximum at λ=578 nm in toluene which corresponds nicely with two previously studied di‐*N*‐arylated DPPs possessing 5,7‐bis(*tert*‐butyl)benzofuranyl substituents (λ=578 and 588 nm in CH_2_Cl_2_).[^24^, ^25^] In stark contrast, DPP **6**, which has the most extended π‐conjugated skeleton, shows a vibronic progression with maxima at λ=504, 545, 595, and 657 nm. Increasing the solvent polarity stepwise, that is, going from toluene via anisole and chlorobenzene to benzonitrile, results in a limited solvatochromatic red‐shift for dyes **3**–**6**. This is accompanied by a steady decrease in the extinction coefficients (Figures S1–S4).

### Steady‐State Fluorescence Spectroscopy

**Figure 3:**
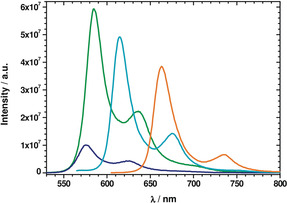
Room‐temperature fluorescence spectra of **3** (dark blue), **4** (green), **5** (pale blue), and **6** (orange) in toluene (3×10^−6^ m) with excitation wavelengths of λ=515, 515, 550, and 590 nm, respectively.

Next, we set our focus on the excited states (ES) by means of steady‐state fluorescence spectroscopy. In toluene, the fluorescence spectra of **3**–**6** are mirror‐images of the absorption spectra, with maxima in the range of λ=575 to 735 nm, and similar vibronic progressions. The Stokes shifts are less than 10 nm (Figures 2 and 3). In a similar manner to the absorption features, the fluorescence maxima of **3**–**6** depend on the size of the π‐conjugated system and shift to longer wavelengths as a function of size (Tables 1 and 2). The fluorescence quantum yields (Φ_fl_) also increase in the following order: **3**<**4**<**5**<**6**, that is, the higher the rigidity of the fused system, the larger is Φ_fl_. The Φ_fl_ value is markedly larger for π‐expanded DPPs **5** and **6**, reaching 39 % and 88 % in nonpolar solvents, respectively (Table 1). These fluorescence quantum yields are comparable to those measured for previously reported π‐expanded DPPs bearing two benzofuran units, but lacking two arene rings and nitro groups.^[18a]^ Our observations suggest that no significant quenching stems from the nitro groups.

**Table 2:** Fluorescence quantum yields (%) of dyes **3**–**6** in different solvents.^[a]^

	Toluene	Anisole	Chlorobenzene	Benzonitrile
**3**	6	0.6	0.5	0.4
**4**	35	0.5	0.3	0.2
**5**	39	1.8	2.9	0.6
**6**	88	58	59	3.2

[a] The fluorescence references used for determining the quantum yields are Rhodamine B for **3** and **4**, Sulforhodamine 101 for **5**, and Nile Blue for **6**.

The absorption and fluorescence maxima of dyes **3**–**6** were hardly varied with the dielectric constants of the solvents, whereas the Φ_fl_ values varied significantly (Table 2, Figures S5–S8). The Φ_fl_ values of DPPs **3**–**5** are highest by far in toluene. Only a slight increase in the polarity, for example by going from toluene to anisole, leads to a strong decrease in the Φ_fl_ value. A further increase in polarity to chlorobenzene and to benzonitrile leads to even lower quantum yields, with values of less than 1 %. However, the spectral shape of the fluorescence remains largely unchanged in the different solvents, thus excluding discernable charge‐transfer contributions in the fluorescent decay.

DPP **6** is a notable exception, as a change from toluene to anisole or chlorobenzene only reduces the fluorescence quantum yield from 88 % to 58 %. It is only in benzonitrile that the drop is significant, and quantum yields as low as 3.2 % are observed (Figures S5–S8). The fact that planarization of the chromophore‐bearing nitroaryl groups makes the emission intensity of the π‐system less susceptible to increasing solvent polarity has already been observed by us when studying dinitropyrrolo[3,2‐*b*]pyrroles.^[28]^

### Time‐Correlated Single‐Photon Counting (TCSPC)

Time‐resolved fluorescence measurements with dyes **3**–**6** were carried out in toluene as well as in benzonitrile. In toluene, all lifetimes are best described by monoexponential decay rates, with values around 4 ns. Significant differences were seen in benzonitrile. Here, the fluorescence lifetimes are less than 200 ps (Table 3). Our findings are in good agreement with the steady‐state fluorescence experiments (see above). From these results, we hypothesize that an additional deactivation pathway, namely population of a structurally relaxed state with charge‐transfer implications, dominates (see below) over fluorescence or intersystem crossing in the more polar solvents.

**Table 3:** TCSPC lifetimes at each respective excitation and emission wavelength in toluene and benzonitrile.

		Toluene		Benzonitrile	
**3**	λ_Exc_=520 nm	1.8 ns	λ_Em_=575 nm	<0.2 ns	λ_Em_=575 nm
**4**	λ_Exc_=530 nm	4.2 ns	λ_Em_=590 nm	<0.2 ns	λ_Em_=590 nm
**5**	λ_Exc_=555 nm	4.1 ns	λ_Em_=620 nm	<0.2 ns	λ_Em_=620 nm
**6**	λ_Exc_=595 nm	3.7 ns	λ_Em_=670 nm	<0.2 ns	λ_Em_=670 nm

### Electrochemistry

**Figure 4:**
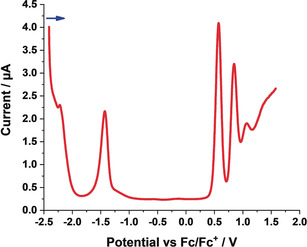
Differential pulse voltammogram of **3** in argon‐saturated dichloromethane (1.5×10^−3^ m) containing 0.1 m TBAPF_6_ at 0 °C. The blue arrow indicates the scan direction.

Electrochemical studies on **3**–**6** were performed in argon‐saturated dichloromethane, containing 0.1 m tetrabutylammonium hexafluorophosphate (TBAPF_6_) as the supporting electrolyte, at 0 °C. For DPP **3**, we observe two oxidations at +0.57 and +0.85 V versus Fc/Fc^+^ and two reductions at −1.43 and −2.22 V versus Fc/Fc^+^ (Figure 4; Table 4). A similar pattern is seen for **4**, with oxidations at +0.84 and +1.09 V versus Fc/Fc^+^ and reductions at −1.17 and −1.45 V versus Fc/Fc^+^ (Figure S9; Table 4). However, for **5** and **6**, three reductions rather than two are observable in addition to the two oxidations (Figures S10 and S11; Table 4). We hypothesize that the additional reduction for **5** and **6**, which are fully conjugated DPPs, are attributed to their extended π‐systems. The electrochemical gaps range from 1.83 eV for **6** to 2.01 eV for **4** (Table 4) and match quite well with the optical gaps derived from steady‐state absorption experiments.

**Table 4:** Oxidations, reductions, and electrochemical gaps in dichloromethane, derived from DPV. All values are given versus Fc/Fc^+^.

	Ox_2_ [V]	Ox_1_ [V]	Red_1_ [V]	Red_2_ [V]	Red_3_ [V]	E_gap_ [eV]
**3**	0.85	0.57	−1.43	−2.22	–	2.00
**4**	1.09	0.84	−1.17	−1.45	–	2.01
**5**	0.78	0.53	−1.36	−1.56	−2.02	1.89
**6**	0.83	0.58	−1.25	−1.60	−2.05	1.83

### Spectroelectrochemistry

To get insight into the spectroscopic features of the oxidized and reduced species of **3**–**6**, spectroelectrochemistry was performed in argon‐saturated benzonitrile containing 0.1 m TBAPF_6_ as a supporting electrolyte (Figures S12–S15). Figure S12 shows the differential absorption spectra of **3** in benzonitrile upon applying a potential of +1.2 V (top) and −1.0 V (bottom) to generate the one‐electron oxidized and reduced form of **3**, respectively. The black spectra in Figures S12–S15 represent the reference spectra prior to any applied potential and, therefore, serve as baselines. Upon oxidation of **3** (Figure S12, top), ground‐state bleaching (GSB) at λ=528 and 573 nm (which mirror the absorption spectrum) is discernable. In addition, three characteristic maxima arise at λ=329, 607, and 737 nm. Major changes in the absorption spectrum are also observed upon reduction of **3** (Figure S12, bottom). GSB is observed at λ=527 and 571 nm. Furthermore, two maxima develop at λ=415 and 631 nm. It should be noted that the optical absorption spectra of the oxidized and reduced species of **3**–**6** look quite similar and are comparable to other spectroelectrochemically investigated DPPs.^[10f]^

### First‐Principles Calculations

**Figure 5:**
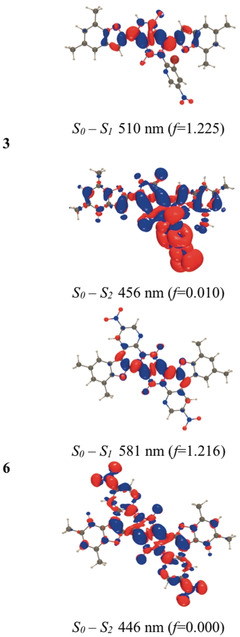
M06‐2X density difference plots for the two lowest lying FC ES in dyes **3** and **6**. The vertical values have no exact experimental counterparts, but the trends in a homologous series are generally similar.^[29]^

We have performed ab initio studies by combining time‐dependent density functional theory (TD‐DFT) and second‐order coupled‐cluster (CC2) calculations including solvent effects (see the Supporting Information). According to DFT, the benzofuran moieties in all compounds are nearly coplanar, with the DPP core in both the S_0_ and S_1_ states. Consistently, the electron density difference plots (Figure 5, see also Figure S32) show that the bright lowest excited state corresponds to a π‐π* excitation delocalized primarily over the four five‐membered rings, with no direct contribution from the NO_2_ groups. In **3** and **4**, the nitropyridine moiety on the N atom is strongly twisted relative to the core in both states (e.g. 72.5° and 72.3° for S_0_ and S_1_, respectively, in **1**). In contrast, perfect planarity is restored in the two “fused” dyes, with true *C_s_* and *C*_2*h*_ minima found for **5** and **6**, respectively. The excited state (ES) is partially delocalized on the additional six‐membered rings, although the nitro groups are again not directly involved. The lack of involvement of the NO_2_ group(s) in the electronic transitions is consistent with the moderate to strong fluorescence of all four DPPs in toluene. The change in the total dipole moment computed for the S_0_→S_1_ transition is always small (no CT effect), and the oscillator strength associated with this transition is very large. Therefore, the steady‐state absorption and emission spectra correspond to this first ES (see also below). In contrast, for all dyes, the S_2_ state has a clear and strong dipolar or quadrupolar CT character—small oscillator strengths and a large change of the dipole moment in the dipolar molecules (Figure 5; see also Figure S32).

In the Franck–Condon (FC) region, this state lies much higher in energy, and is (almost or perfectly) dark, so that it cannot play a role in the steady‐state measurements, where one notices small Stokes shifts, small solvatochromic changes of the transition wavelengths, and strong vibronic couplings in all solvents. The computational studies suggest that **3**, after excitation, reaches (S_1_)_LE_, but cannot directly deactivate to the CT state. Instead, it undergoes solvent and structural relaxation before undergoing intersystem crossing (see below).

In Table 5 we give the vertical and 0‐0 energies determined with a composite CC2/TD‐DFT approach. The 0‐0 energies can be straightforwardly compared with the experimentally determined crossing point between the absorption and emission.^[30^ The absolute values are within ca. 0.15 eV of the experimental data, typical for such a level of theory. More impressive is the agreement in the series: When going from **3** to **4**, only a modest bathochromic displacement of −0.04 eV is computed, in line with −0.03 eV determined by experiments. This shift is due to the inductive effect of the additional pyridine ring(s). Consistent with the experiments, much larger red‐shifts are obtained for the fused DPPs, with displacements of the 0‐0 energies determined to be −0.17 eV and −0.31 eV for **5** and **6**, respectively, relative to **3**. The experimental values are −0.14 and −0.29 eV, respectively.

**Table 5:** Vertical transition energies and wavelengths determined for the absorption and fluorescence processes in dyes **3**–**6**.^[a]^

	Absorption			Fluorescence			0‐0 energies		
	Δ*E* [eV]	λ [nm]		Δ*E* [eV]	λ [nm]		Δ*E* [eV]	λ [nm]	Δ*E* [nm] Expt.
**3**	2.430	510		2.139	580		2.034	610	571
**4**	2.375	522		2.100	590		1.995	622	580
**5**	2.294	541		1.999	620		1.881	659	612
**6**	2.133	581		1.851	670		1.724	719	660

[a] On the right‐hand‐side, the 0‐0 energies obtained theoretically and measured experimentally are also given. All results used a composite CC2/TD‐M06‐2X approach and were obtained in toluene.

**Figure 6:**
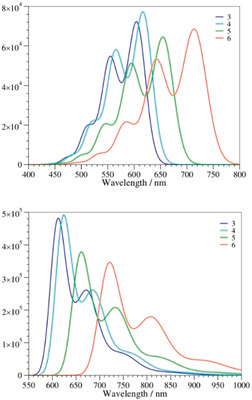
Computed vibrationally resolved spectra for **3**–**6** in toluene: absorption (top) and emission (bottom).

Experimentally, both the absorption and fluorescence spectra show a clear vibronic progression. We accounted for vibronic couplings as described in the Supporting Information.^[30]^ As shown in Figure 6, there is a clear match between theory and experiment in terms of band shapes for both the absorptions and emissions, with a clear second maximum and a shoulder at shorter (longer) wavelengths for the absorptions (fluorescence). This confirmed the presence of similar strong vibronic couplings in all the compounds. For example, in **6**, the second absorption band is mainly due to two ES vibrations at $\tilde{\nu }$ =1536 and 1705 cm^−1^, whereas in the case of emission, only one mode at $\tilde{\nu }$ =1669 cm^−1^ can explain the presence of a second fluorescence band. All these vibrations correspond to so‐called “ECC stretching” modes, in which the nature of the single/double bonds in the conjugated core of the dye are modified by the vibrations. The separation between the main and second maxima is, therefore, about $\tilde{\nu }$ =1700 cm^−1^ or 0.21 eV, both experimentally and theoretically.

**Figure 7:**
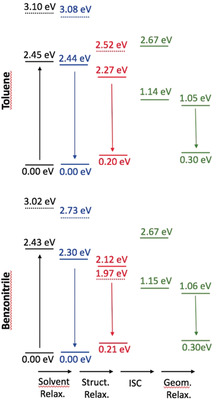
Relative ωB97X‐D/6‐311+G(2dp) energy levels determined for *N*‐arylated DPP **3** in toluene (top) and benzonitrile (bottom) with the LR+cLR solvent model, taking the FC point as reference. The full (dashed) lines correspond to the S_1_ (S_2_) state of dominant local (CT) character. The black values correspond to the FC point, blue data to the relaxed solvent, red to relaxed solvent and structural parameters, whereas the green values are for both the relaxed and unrelaxed triplet ES.

**Figure 8:**
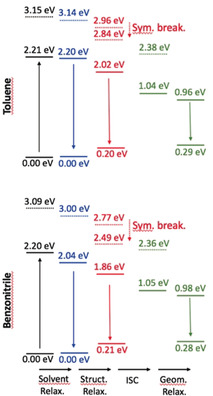
Relative wB97X‐D/6‐311+G(2dp) energy levels determined for π‐expanded DPP **6** in toluene (top) and benzonitrile (bottom). See caption of Figure 7 for more details.

To further investigate the various ES processes, we have performed additional calculations on the relaxation effects (see the Supporting Information for details). As the S_2_ state has a very strong CT character (see above), we have been careful to use a range‐separated hybrid and to account for state‐specific solvation effects. An illustration of the findings is given in Figures 7 and 8 for DPPs **3** and **6** (see Figures S34–S37 for all compounds). First, as expected, the (S_2_)_CT_ state undergoes a symmetry‐breaking process (the symmetric structure becomes unstable in the (S_2_)_CT_ state after relaxation), and it can be seen that its energy becomes closer to that of the (S_1_)_LE_ state relaxed in the same way, especially in the case of **3** and **4** in benzonitrile, for which this CT state becomes even lower in energy. Nevertheless, we underline that the irradiation wavelength used in the experimental set‐up (see below) corresponds to the excitation of the dipole‐allowed S_1_ state, so that a fully relaxed S_2_ state (as shown in Figure 7) cannot be reached directly. Secondly, the solvent relaxation has a tiny effect in toluene (ca. −0.01 eV), whereas it results in a significant red‐shift for the lowest excited‐state in benzonitrile (ca. −0.15 eV). The subsequent structural relaxation is significant in both solvents, thus making the structurally relaxed S_1_ state 0.34 eV below the original (FC) S_1_ state for **6** in benzonitrile, compared with a much smaller difference of 0.19 eV in toluene for the same dye. From Figure 7, we derive that only the lowest triplet state is accessible for ISC, as T_2_ is higher in energy than S_1_. In other words, only one triplet should play a role in the relaxation process. Similar results were obtained for π‐expanded DPP **5**.

### Transient Absorption Spectroscopy

Femtosecond transient absorption (fsTA; 0–7500 ps) and nanosecond transient absorption (nsTA; 1 ns −400 μs, 450 nJ) measurements helped to elucidate the excited‐state features and kinetics of DPPs **3**–**6**.^[31]^ They were carried out in toluene and benzonitrile with excitation at λ=550 nm (fsTA: *E*=500 nJ; nsTA: *E*=450 nJ). Evaluation of the fsTA and nsTA data was carried out with a combination of multiwavelength and GloTarAn target analyses. Focusing first on the fsTA measurements in toluene, **3**–**6** feature the spontaneous formation of the singlet excited state S_1_. For example, in **3** S_1_ includes broad maxima at λ=450 and 955 nm and minima at λ=525, 572, and 630 nm corresponding to the ground‐state bleaching (GSB) and stimulated emission (SE), respectively. These features are replaced, on one hand, by a long‐lived singlet excited state (S_1_)_SOL_ feature after solvent reorganization has taken place, and, on the other hand, a short‐lived singlet excited state (S_1_)_STR_ after structural relaxation has taken place. The former is caused by the reduction of the solvation energy of the excited state serving as the main driving force, while the latter infers reorganization of the potential energy surface and is likely to possess a sizeable contribution from the redistribution of charge density. The formation of (S_1_)_STR_ induces a 8 nm red‐shift of the GSB at λ=630 nm and a 28 nm blue‐shift and band‐narrowing of the maximum at λ=955 nm. In contrast, (S_1_)_SOL_ causes a 4 nm blue‐shift of the GSB and a 28 nm blue‐shift and narrowing of the features at λ=630 and 970 nm, respectively.

Similar GSBs and SEs are found for DPPs **4**–**6** in the visible region (Figures S16–S22). These shifted in line with the respective steady‐state absorption and fluorescence measurements. Strong differences are, however, noted in the NIR region.

DPP **4** still features a broad maximum at about λ=1045 nm. Expanded DPPs **5** and **6** feature a narrower fine‐structure, with maxima at λ=894 and 1142 nm for **5** and at λ=925 and 1152 nm for **6**. We attribute these differences, on the one hand, to the flexible nature of the nonbridged substituents of the DPP core in **3** and **4**. The immediate consequence is a broader feature in the NIR region. On the other hand, the rigidity in **5** and **6** leads to a narrow S_0_→S_1_ transition and fine‐structure of the NIR bands. Similar shifts were noted for the remaining transitions. We postulate that for **3**–**6** in toluene, a parallel population of (S_1_)_SOL_ and (S_1_)_STR_ from S_1_ exists, since the energy levels are very close. This was independently confirmed by electrochemical measurements and theoretical calculations. Similar (S_1_)_SOL_ and (S_1_)_STR_ energies enable significant mixing and, in turn, strong and long‐lived fluorescent deactivation (see above) in toluene.

**Figure 9:**
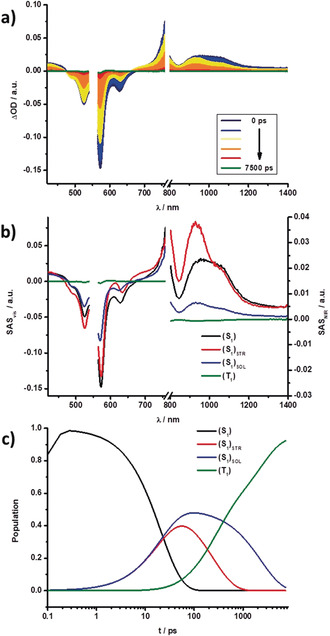
a) Differential absorption spectra obtained from fsTA experiments (λ=550 nm) of **3** in toluene with several time delays between 0 and 7500 ps at rt. b) Species‐associated spectra of the transient absorption data of **3** shown in (a), with the initially formed singlet excited state S_1_ (black), structurally relaxed singlet excited state (S_1_)_STR_ (red), solvent‐reorganized singlet excited state (S_1_)_SOL_ (blue), and the triplet excited state T_1_ (green) obtained from GloTarAn. c) Relative population kinetics of the respective states.

**Figure 10:**
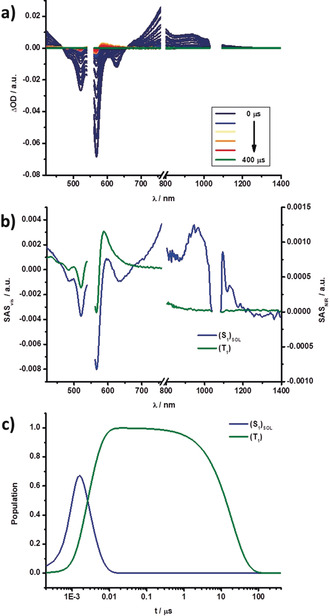
a) Differential absorption spectra obtained by nsTA experiments (λ=550 nm) of **3** in toluene with several time delays between 0 and 400 μs at rt. b) Species‐associated spectra of the transient absorption data of **3** shown in (a), with the solvent‐reorganized singlet excited state (S_1_)_SOL_ (blue), and the triplet excited state T_1_ (green) obtained from GloTarAn. c) Relative population kinetics of the respective states.

Both states, in turn, decay into a fourth species, which we assign to a triplet excited state (T_1_), by intersystem crossing (ISC). Its growth and decay on the microsecond timescale can be easily followed in the respective nsTA measurements. The spin‐forbidden ISC and, in turn, inefficient singlet to triplet excited‐state transformation results in weak and rather poorly resolved triplet excited‐state features in **3**–**6**. These features consist of GSB and a maximum, which is, for example at λ=590 nm for **3**. In all cases, T_1_ has the same overall shape. Figures 9 and 10 showcase the fsTA and nsTA spectra, respectively, of **3** in toluene.

Changing the solvent to benzonitrile led to significant changes (Figures S23–S30). Importantly, the initial S_1_ population remains the same, but the subsequent deactivation cascade changes. In particular, (S_1_)_SOL_, which is populated initially, transforms into (S_1_)_STR_. A significant change in lifetimes is also noted. (S_1_)_SOL_ decays in benzonitrile on the picosecond timescale. This is also reflected in the TCSPC measurements, which, in turn, helped to identify (S_1_)_SOL_ in the solvent despite the drastically different lifetimes. In contrast, the (S_1_)_STR_ state appeared to be stabilized in benzonitrile, as a result of a lowering of its energy level. The parallel deactivation is replaced by a sequential deactivation because of a lower (S_1_)_STR_ energy. A potential mixing with (S_2_)_STR_ cannot be ruled out. The last steps, namely ISC to T_1_ and recovery of S_0_, remained unchanged. A complete overview of the respective lifetimes in each solvent is given in Table 6.

**Table 6:** fsTa and nsTA lifetimes of dyes **3**‐**6** in toluene and benzonitrile.

		*τ*(S_1_)	*τ*(S_1_)_STR_/Tol	*τ*(S_1_)_SOL_	*τ*(S_1_)_STR_/BN	*τ*(T_1_)
toluene	**3**	20.78 ps	244.95 ps	2.35 ns	–	19.19 μs
	**4**	3.68 ps	119.29 ps	1.49 ns	–	21.14 μs
	**5**	1.38 ps	269.38 ps	2.86 ns	–	21.89 μs
	**6**	8.60 ps	228.41 ps	3.93 ns	–	69.94 μs
						
benzonitrile	**3**	3.14 ps	–	8.43 ps	19.19 ns	133.86 μs
	**4**	5.19 ps	–	13.46 ps	23.38 ns	13.58 μs
	**5**	8.60 ps	–	24.30 ps	22.03 ns	117.56 μs
	**6**	3.17 ps	–	143.00 ps	13.82 ns	4.16 μs

## Conclusion

Herein, we have opened a new avenue towards structurally unique, X‐shaped, π‐expanded diketopyrrolopyrroles by using intramolecular, direct arylation as a pivotal step. Our strategy is based on the presence of four essential functional groups in the structure: two activating electron‐withdrawing groups, one fluorine atom, and one bromine atom. More importantly, all of them must be in a suitable arrangement. A two‐step process transforms the parent DPP into structures with up to 10 fused rings. As a consequence, the absorptions are bathochromically shifted to the deep‐red region and strong red emission is seen, despite the presence of NO_2_ groups. The latter are inactive in the S_0_→S_1_ electronic transition, even when fused to the DPP core, thereby resulting in strong fluorescence in nonpolar solvents with quantum yields ranging from 6 % to 88 % and lifetimes on the order of several nanoseconds. Although the quantum yields are between 0.4 % and 3.2 % and the lifetimes are on the order of tens of picoseconds in polar solvents, the NO_2_ groups have no impact. As such, the presence of NO_2_ groups fails to impose any charge‐transfer character on the S_1_ state. This character is only present in the S_2_ state, which was, however, not probed experimentally. Instead, time‐resolved techniques and computational studies show that internal conversion and intersystem crossing to afford a structurally relaxed (S_1_)_STR_ state with a sizeable charge‐transfer contribution and a T_1_ state, respectively, are responsible for the fluorescence quenching in both *N*‐nitroaryl‐DPPs and π‐expanded DPPs. These results are not only of theoretical significance in that they provide new insights into factors stemming from NO_2_ groups and influencing the fluorescent properties, but they may also open the door for the design of nitroaromatic compounds possessing strong and tunable fluorescence. The perspective applications of these novel π‐expanded DPPs include, but are not limited to, low band gap copolymers,^[19a]^ NIR organic field‐effect transistors,[^18c^, ^19b^] solution‐processed solar cells,^[19c]^ and NIR organic light‐emitting diodes for visible‐light communication.^[19e]^

## Conflict of interest

The authors declare no conflict of interest.

## Supporting information

As a service to our authors and readers, this journal provides supporting information supplied by the authors. Such materials are peer reviewed and may be re‐organized for online delivery, but are not copy‐edited or typeset. Technical support issues arising from supporting information (other than missing files) should be addressed to the authors.

Supplementary
