# Supplementary material for: How To Make Nitroaromatic Compounds Glow: Next‐Generation Large X‐Shaped, Centrosymmetric Diketopyrrolopyrroles
Source: Angew Chem Int Ed Engl. 2020 Jul 10;59(37):16104–13. doi: 10.1002/anie.202005244 (PMC7689858; doi:10.1002/anie.202005244)
Supplement: Supplementary file 1 — Supplementary [file ANIE-59-16104-s001.pdf]

Angewandte Chemie
Eine Zeitschrift der Gesellschaft Deutscher Chemiker

Supporting Information

How To Make Nitroaromatic Compounds Glow: Next-Generation Large X-Shaped, Centrosymmetric Diketopyrrolopyrroles

Kamil Skonieczny[+], Ilias Papadopoulos[+], Dominik Thiel[+], Krzysztof Gutkowski, Philipp Haines, Patrick M. McCosker, Adèle D. Laurent, Paul A. Keller, Timothy Clark, Denis Jacquemin, Dirk M. Guldi,* and Daniel T. Gryko**

anie_202005244_sm_miscellaneous_information.pdf

Author Contributions

K.S. Investigation: Lead; Visualization: Equal; Writing - Original Draft: Equal; Writing - Review & Editing: Supporting
I.P. Investigation: Equal; Visualization: Equal; Writing - Original Draft: Supporting
D.T. Investigation: Equal; Visualization: Supporting; Writing - Original Draft: Equal
K.G. Investigation: Supporting; Writing - Original Draft: Supporting
P.H. Investigation: Supporting; Visualization: Supporting
P.P. Investigation: Equal
A.L. Investigation: Equal
P.K. Investigation: Equal; Visualization: Supporting; Writing - Original Draft: Equal
T.C. Supervision: Equal; Writing - Original Draft: Equal
D.J. Investigation: Supporting; Supervision: Equal; Writing - Original Draft: Equal; Writing - Review & Editing: Equal
D.G. Supervision: Lead; Writing - Original Draft: Lead; Writing - Review & Editing: Supporting.

Table of Contents

1. General Methods S2-S3
2. Experimental Procedures S4-S7
3. Spectral Data S8-S11
4. Photophysical Data S12
    4.1 Steady-State Absorption Spectroscopy S12-S14
    4.2 Steady-State Fluorescence Spectroscopy S15-S18
    4.3 Electrochemistry S19-S20
    4.4 Spectroelectrochemistry S21-S24
    4.5 Transient Absorption Spectroscopy S25-S40
5. Computational Details S41-S48

1. General methods

All chemicals were used as received unless otherwise noted. All reported ^1H NMR and ^{13}C NMR spectra were recorded on 500 MHz spectrometer. Chemical shifts (δ ppm) were determined with TMS as the internal reference; *J* values are given in Hz. Mass spectra were obtained via EI MS. For HRMS measurements both quadruple and TOF mass analyzer types were used. UV–Vis and fluorescence spectra were recorded in toluene, chlorobenzene, anisole and benzonitrile. Chromatography was performed on silica (Kieselgel 60, 200–400 mesh). DPP **2** was prepared according to the literature procedures.[1]

Steady-state measurements were performed on a PerkinElmer Lambda2 UV/Vis two-beam spectrophotometer with a slit width of 2 nm and a scan rate of 240 nm•min^{-1} for absorption assays, and a Horiba Jobin Yvon FluoroMax-3 spectrometer with a slit width of 2 nm for excitation and emission and an integration time of 0.5 s in a wavelength range of 500–1000 nm for fluorescence assays, respectively. Fluorescence quantum yields (Φ_{fl}) were obtained using Rhodamine B (Φ_{fl} = 70% in EtOH) for **3** and **4**, Sulforhodamine 101 (Φ_{fl} = 90% in EtOH) for **5**, and Nile Blue (Φ_{fl} = 27% in MeOH) for **6** as standards, respectively. Time correlated single photon counting (TCSPC) measurements were carried out on a Horiba Jobin Yvon FluoroLog3 emission spectrometer utilizing a Hamamatsu MCP photomultiplier (R3809U-58), in order to obtain the fluorescence lifetimes. A laser diode (NanoLED-405L, 530 nm, pulse width ≤200 ps, maximum of repetition rate 100 kHz) was utilized for excitation.

For the differential pulse voltammetry (DPV) assays a three electrode cell configuration with a platinum working electrode, a platinum mesh counter electrode, and a silver wire quasi reference electrode were utilized. The measurements were performed in argon saturated dichloromethane at 0 °C, a respective DPP concentration of 1.5 x 10^{-3} M, and using $TBAPF_6$ (0.1 M) as supporting electrolyte. All the voltammograms were corrected against a Fc/Fc^+ redox couple as internal standard.

Spectroelectrochemical assays were performed on a home-made three-necked cell consisting of a platinum mesh counter electrode, a silver wire quasi-reference electrode, and a platinum

[1] M. Grzybowski, E. Glodkowska-Mrowka, T. Stoklosa, D. T. Gryko, *Org. Lett.* **2012**, *14*, 2670.

grid as working electrode. Measurements were done in argon saturated benzonitrile at room temperature, using $TBAPF_6$ (0.1 M) as supporting electrolyte. Potentials were adjusted with a Metrohm PGStat 101 (2 min hold time) and recorded using a Cary 5000 double beam spectrometer from Varian with the WinUV software. Femto- (fsTA) and nanosecond (nsTA)

Transient absorption assays on the femto- to nanosecond timescale (fsTA) were performed using an amplified CPA-2110 titanium:sapphire laser (1kHz; 150 fs pulse width; 500 nJ laser energy) from Clark-MXR Inc. Measurements on the nano- to microsecond timescale (nsTA) were performed on the EOS SYSTEM from Ultrafast Systems operated with a 1 kHz pump laser (450 nJ laser energy). Excitation was at 550 nm wavelength. A 2 kHz continuous white light fibre laser was used for probing. Analysis of the fsTA and nsTA data was executed using a combination of multiwavelength, through the OriginPro software, and target analysis, through the GloTarAn software, respectively.

2. Experimental procedures

2-(3-Bromo-5-nitropyridin-2-yl)-3,6-bis(5,7-di-*tert*-butylbenzofuran-2-yl)-2,5-dihydropyrrolo[3,4-*c*]pyrrole-1,4-dione (3)

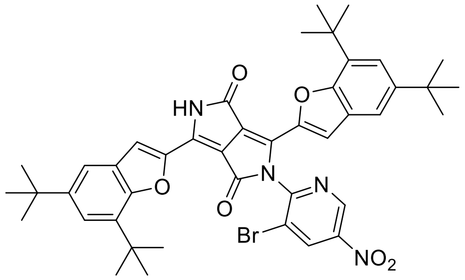

A mixture of 3-bromo-2-chloro-5-nitropyridine (**1**) (144 mg, 0.607 mmol), KF (70 mg, 1.2 mmol) in 200 mL of DMF was stirred at 100 °C for 3h. The mixture was then cooled to 70 °C. DPP **2** (300 mg, 0.506 mmol), K_2CO_3 (70 mg, 0.51 mmol) were added and reaction was stirred at 70 °C for 12h. The solvent was evaporated under vacuum. The solid residue was dissolved in CH_2Cl_2 (500 mL) and the solution was washed with water and brine. Organic layer was dried over $MgSO_4$ and the solvent was removed under reduced pressure. A mixture of unreacted substrate and mono *N*-arylated, bis-*N*,*N*-arylated derivatives was obtained. The target product was purified by silica gel chromatography using hexanes / ethyl acetate (15/1) solution as eluent. Crystallization from ethyl acetate / ethanol afforded analytically pure compound.

Yield: 120 mg (30%). Red solid; m.p. 285-286 ºC; ^{1}H NMR (500 MHz, $CDCl_3$) δ 9.36 (d, J = 2.5 Hz, 1H), 8.93 (d, J = 2.5 Hz, 1H), 8.59 (s, 1H), 8.43 (s, 1H), 8.20 (s, 1H), 7.55 (d, J = 1.8 Hz, 1H), 7.51 (d, J = 1.8 Hz, 1H), 7.44 (d, J = 1.8 Hz, 1H), 7.29 (d, J = 1.8 Hz, 1H), 1.55 (s, 9H), 1.38 (s, 9H), 1.34 (s, 9H), 1.01 (s, 9H); ^{13}C NMR (125 MHz, $CDCl_3$) δ 160.8, 159.0, 153.7, 152.7, 152.5, 147.5, 147.4, 144.1, 143.6, 143.4, 143.1, 137.5, 134.4, 133.7, 133.1, 132.4, 128.9, 128.5, 123.5, 123.1, 121.4, 117.2, 116.7 (2 signals), 116.2, 111.3, 107.2, 35.0, 34.9, 34.6, 33.8, 31.7, 31.6, 30.0, 29.4; HRMS (EI): m/z calcd for $C_{43}H_{45}BrN_4O_6$ [$M^{\cdot+}$] 792.2522, found 792.2526.

2,5-Bis(3-bromo-5-nitropyridin-2-yl)-3,6-bis(5,7-di-*tert*-butylbenzofuran-2-yl)-2,5-dihydropyrrolo[3,4-*c*]pyrrole-1,4-dione (4)

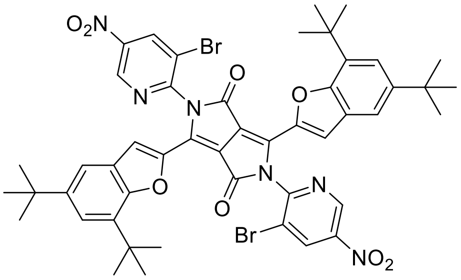

A mixture of 3-bromo-2-chloro-5-nitropyridine (**1**) (950 mg, 4.00 mmol) and KF (465 mg, 8.00 mmol) in 200 mL of DMF was stirred at 100 °C for 3h. The mixture was then cooled to 70 °C. DPP **2** (593 mg, 1.00 mmol), K_2CO_3 (553 mg, 4.00 mmol) were added and reaction was stirred at 70 °C for 12h. The solvent was evaporated under vacuum. The organic residue was dissolved in 100 mL of EtOAc and filtered to remove inorganic solid. The resulting solution was concentrated to 20 mL and treated with 100 mL of methanol. The obtaining precipitate was filtered off and washed with water and methanol.

Yield: 986 mg (100%). Dark red solid; m.p. = 378-379 ºC; ^{1}H NMR (500 MHz, $CDCl_3$) δ 9.42 – 9.40 (m, 1H), 9.36 – 9.35 (m, 1H), 8.96 (t, *J* = 2.8 Hz, 2H), 8.61 (s, 1H), 8.57 (s, 1H), 7.50 (t, *J* = 1.8 Hz, 2H), 7.31 (dd, *J* = 3.0, 2.0 Hz, 2H), 1.33 (s, 18H), 1.02 (s, 18H); ^{13}C NMR (125 MHz, $CDCl_3$) δ 159.3, 159.2, 153.5, 153.4, 152.8, 147.6 (2 signals), 144.2, 144.1, 143.5 (2 signals), 143.2 (2 signals), 137.6, 137.5, 133.8 (2 signals), 133.5, 128.5, 123.6, 123.5, 121.5, 121.2, 118.3, 118.2, 116.8, 109.3, 109.0, 34.9, 33.9 (2 signals), 31.6, 29.4; HRMS (EI): calcd for $C_{48}H_{46}Br_2N_6O_8$ [$M^{\cdot+}$] 992.1744, found 992.1747.

EDPP 5

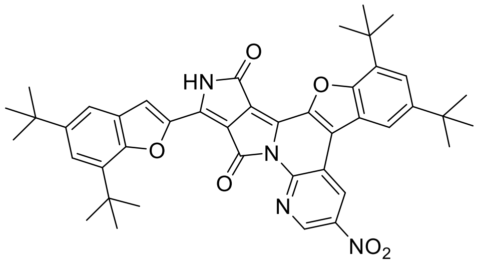

A mixture of *N*-arylated Diketopyrrolopyrrole **3** (100 mg, 0.13 mmol), $Pd(PPh_3)_4$ (14 mg, 0.013 mmol), KOAc (25 mg, 0.25 mmol) in dry toluene (6 ml) was stirred at 100 °C for 24h in Schlenk tube. The mixture was diluted by CH_2Cl_2 (50 ml) and then evaporated with Celite. The target product was purified by silica gel chromatography using hexanes / ethyl acetate (9/1) solution as eluent. Crystallization from ethyl acetate / ethanol afforded analytically pure compound.

Yield: 72 mg (80%). Dark blue solid; m.p. >400 ºC; ^{1}H NMR (500 MHz, $CDCl_3$) δ 9.50 (d, *J* = 2.5 Hz, 1H), 9.18 (d, *J* = 2.6 Hz, 1H), 8.45 (s, 1H), 8.24 (s, 1H), 7.95 (d, *J* = 1.5 Hz, 1H), 7.64 (d, *J* = 1.5 Hz, 1H), 7.54 (d, *J* = 1.7 Hz, 1H), 7.46 (d, *J* = 1.8 Hz, 1H), 1.66 (s, 9H), 1.54 (s, 9H), 1.50 (s, 9H), 1.40 (s, 9H); ^{13}C NMR (125 MHz, $CDCl_3$) δ 160.0, 157.0, 154.9, 152.8, 149.9, 148.9, 147.7, 144.2, 143.5, 142.9, 140.4, 136.3, 135.5, 134.4, 128.9, 126.8, 126.6, 124.7, 124.0, 122.7, 118.9, 117.3, 116.8, 116.2, 114.9, 107.3, 105.3, 35.3, 35.0, 34.9, 34.6, 31.8, 31.7, 29.9, 29.8, 29.7; HRMS (EI): calcd for $C_{43}H_{44}N_4O_6$ [$M^{\cdot+}$] 712.3261; found 712.3264.

EDPP 6

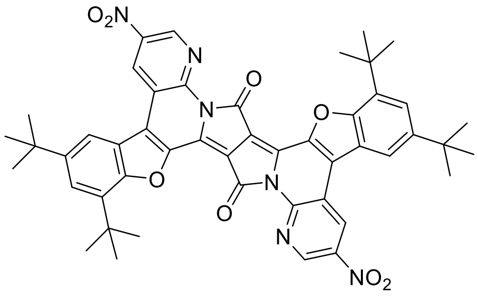

A mixture of *N*-arylated Diketopyrrolopyrrole **4** (448 mg, 0.450 mmol), $Pd(PPh_3)_4$ (52 mg, 0.045 mmol), KOAc (177 mg, 1.80 mmol) in dry toluene (6 ml) was stirred at 100 °C for 24h in Schlenk tube. The mixture was diluted by CH_2Cl_2 (150 ml) and then evaporated with Celite. The target product was purified by silica gel chromatography using CH_2Cl_2 / methanol (99/1) solution as eluent. Crystallization from ethyl acetate / ethanol afforded analytically pure compound.

Yield: 321 mg (86%). Dark blue solid; m.p. >400 ºC; ^{1}H NMR (500 MHz, $CDCl_3$) δ 9.62 (d, *J* = 2.5 Hz, 2H), 9.26 (d, *J* = 2.6 Hz, 2H), 8.00 (d, *J* = 1.6 Hz, 2H), 7.70 (d, *J* = 1.7 Hz, 2H), 1.72 (s, 18H), 1.51 (s, 18H); ^{13}C NMR (125 MHz, $CDCl_3$) δ 156.4, 155.4, 149.7, 149.2, 144.2, 144.1, 140.5, 136.8, 129.7, 127.1, 125.4, 122.6, 116.2, 115.0, 110.0, 103.0, 35.3, 35.1, 31.7, 30.0. HRMS (EI): m/z calcd for $C_{48}H_{44}N_6O_8$ [$M^{\cdot+}$] 832.3221; found 832.3224.

3. Spectral data

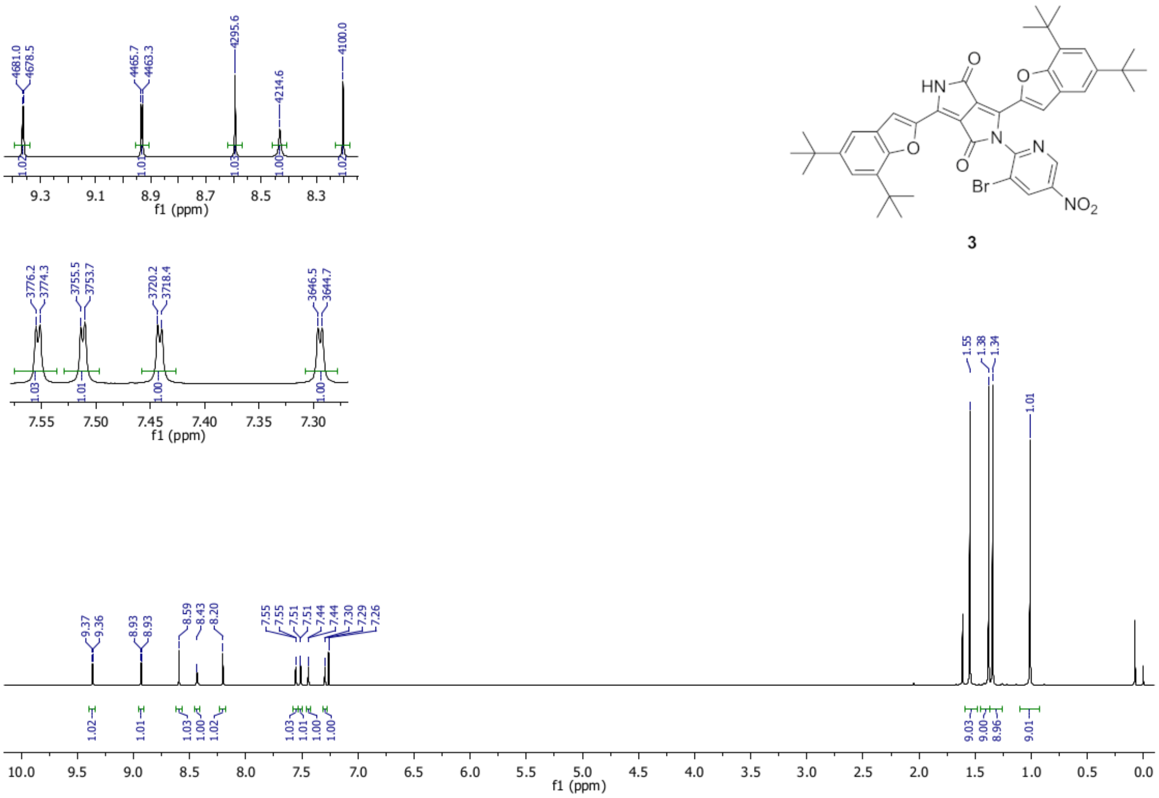

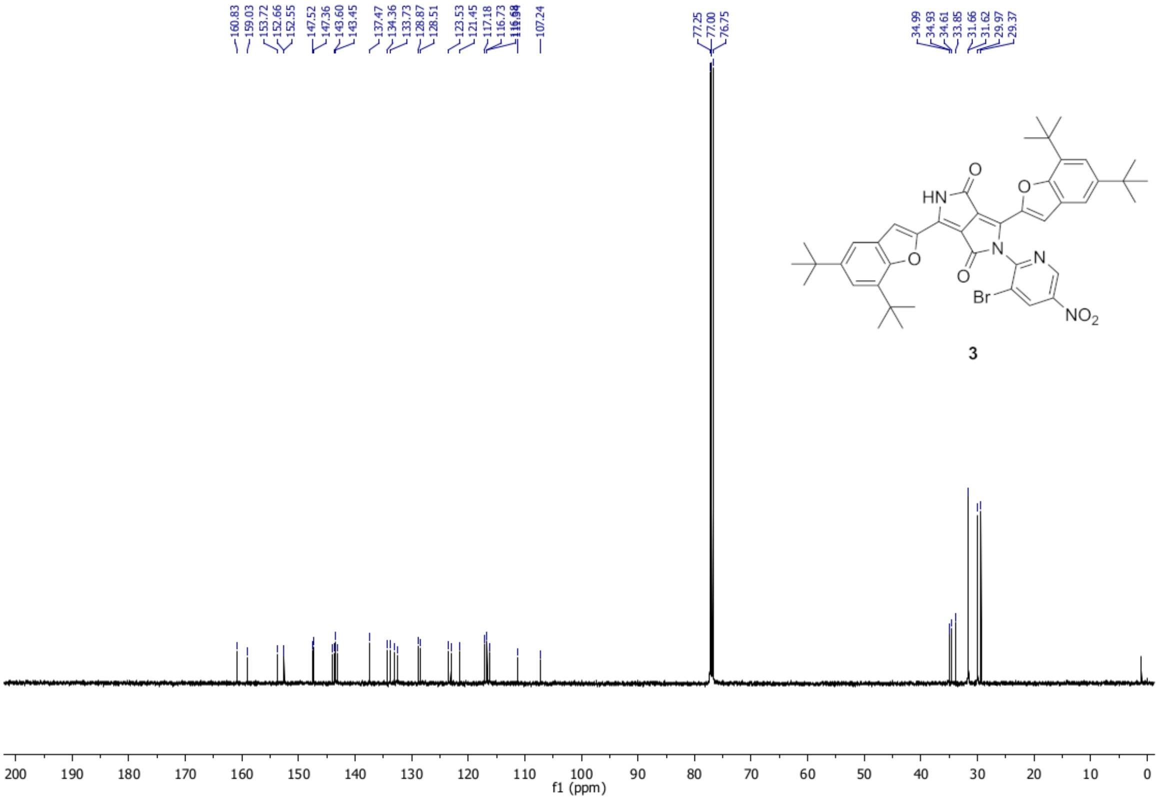

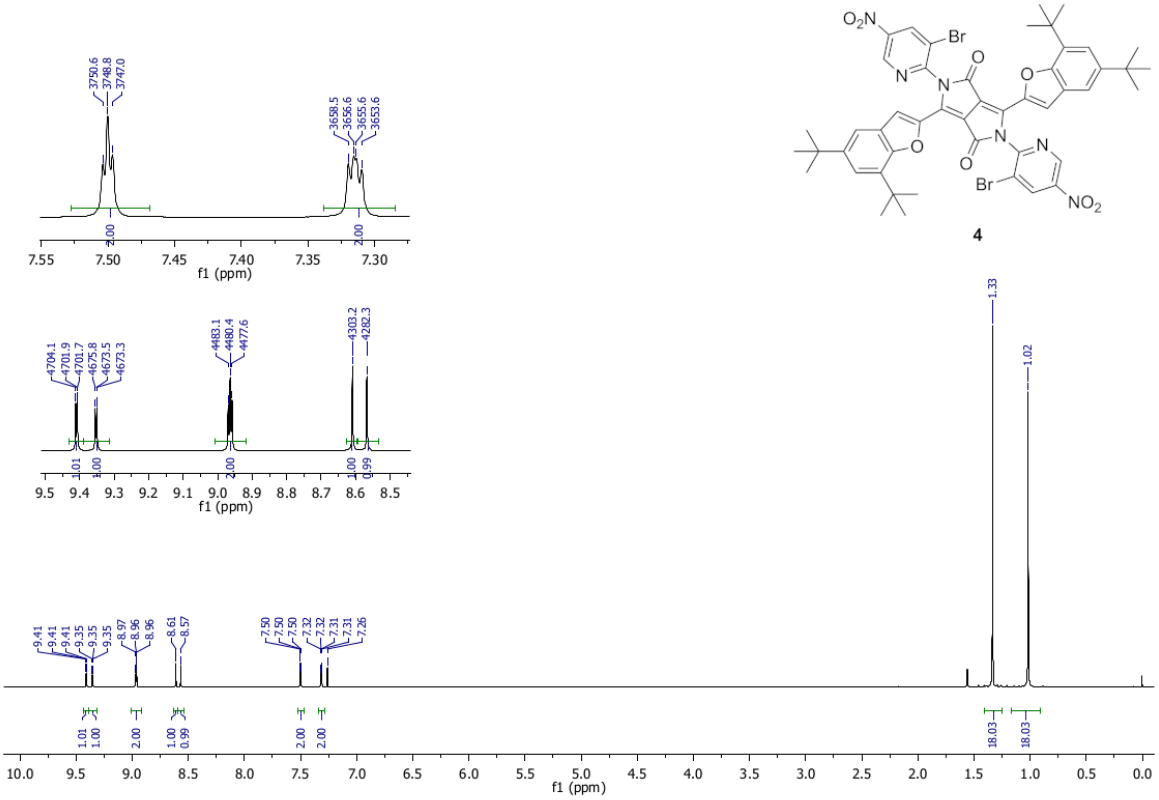

4

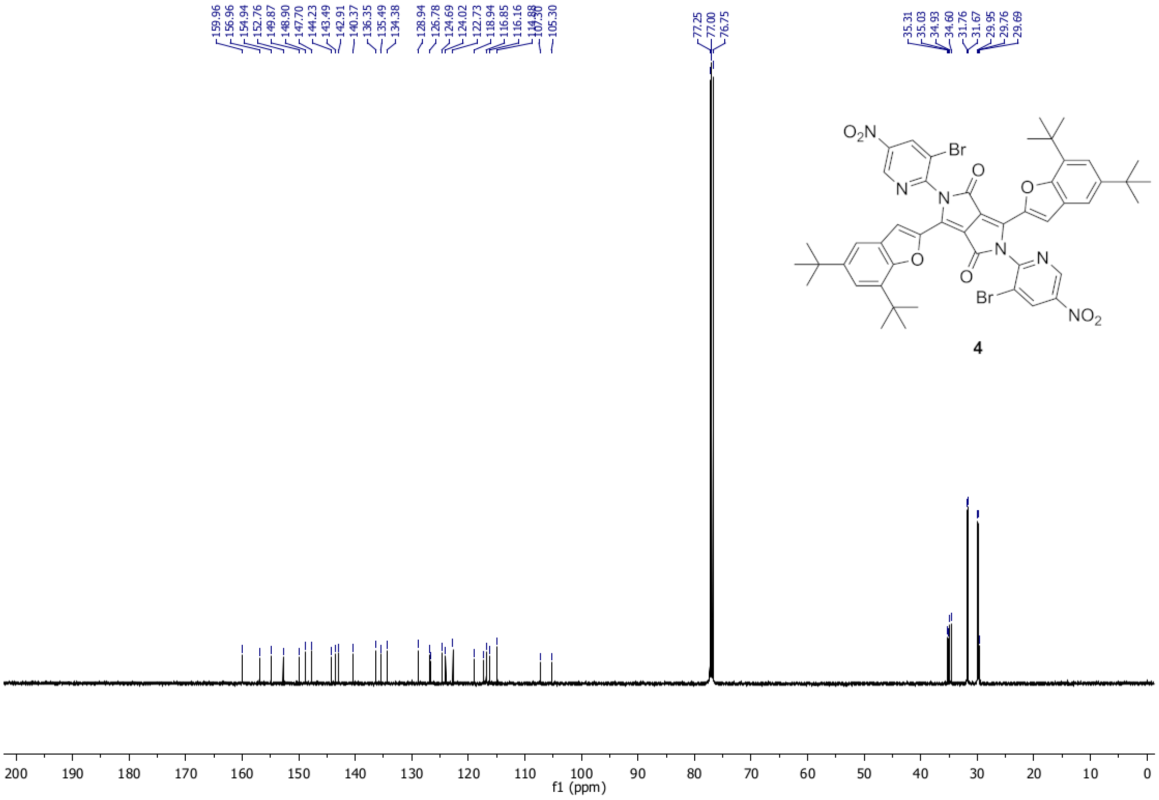

4

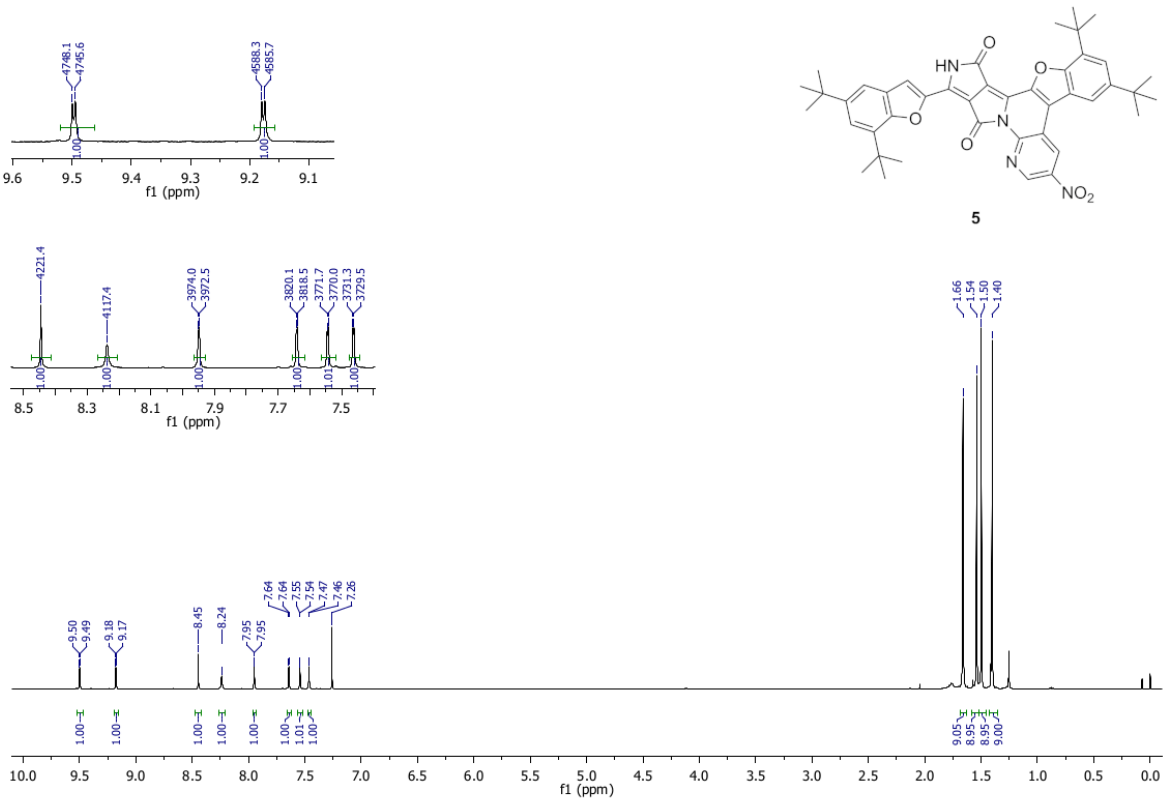
HN
O
N
NO2
f1 (ppm)

5

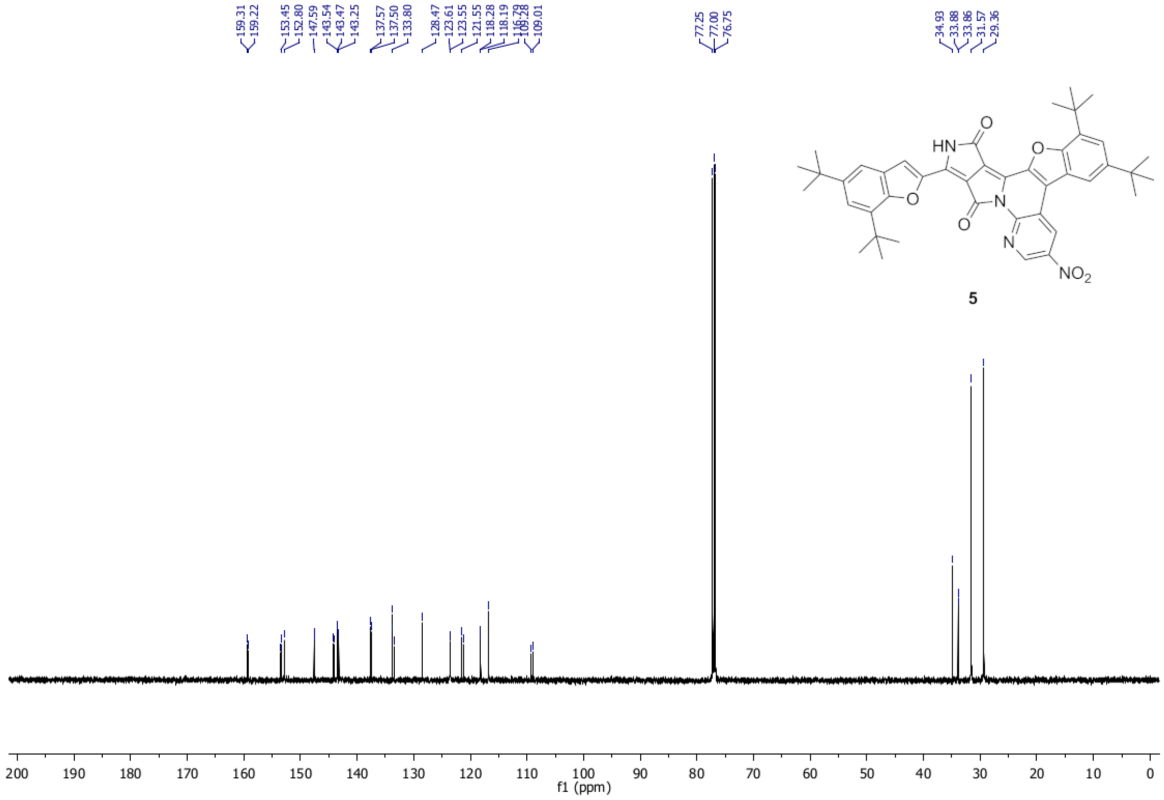
159.31
159.22
153.45
152.80
147.59
143.54
143.47
143.25
137.57
137.50
133.80
128.47
123.61
123.55
121.55
118.28
118.19
109.01
77.25
77.00
76.75
34.93
33.88
33.86
31.57
29.36
HN
O
N
NO2
f1 (ppm)

5

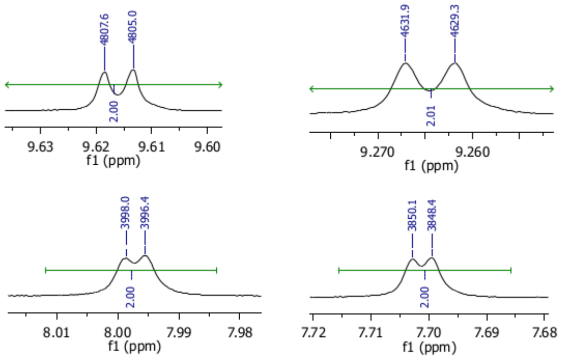
4807.6
4805.0
2.00
9.63 9.62 9.61 9.60
f1 (ppm)
4631.9
4629.3
2.01
9.270 9.260
f1 (ppm)
3998.0
3996.4
2.00
8.01 8.00 7.99 7.98
f1 (ppm)
3850.1
3848.4
2.00
7.72 7.71 7.70 7.69 7.68
f1 (ppm)

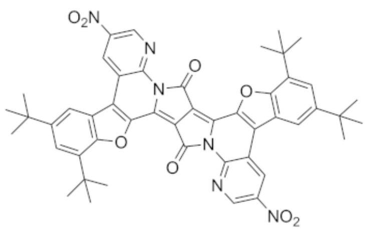
O2N
N
O
O
N
O
O
N
N
NO2

6

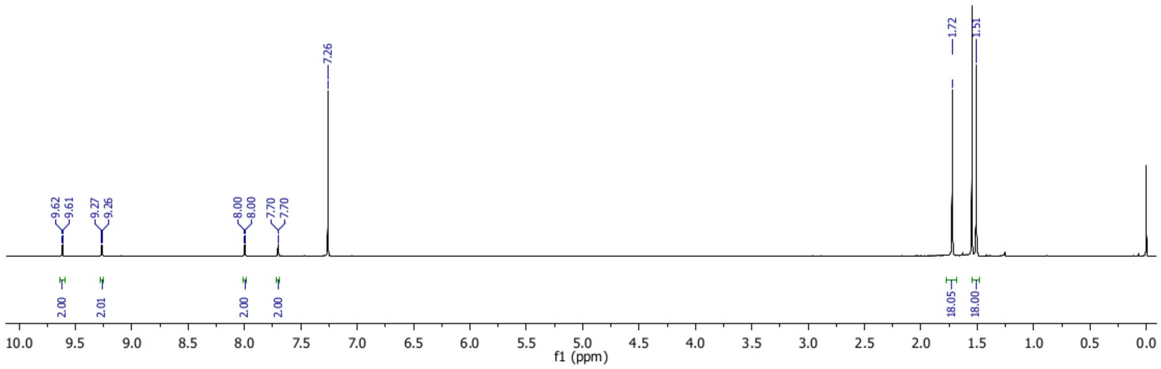
9.62
9.61
9.27
9.26
8.00
8.00
7.70
7.70
7.26
1.72
1.51
2.00
2.01
2.00
2.00
18.05
18.00
10.0 9.5 9.0 8.5 8.0 7.5 7.0 6.5 6.0 5.5 5.0 4.5 4.0 3.5 3.0 2.5 2.0 1.5 1.0 0.5 0.0
f1 (ppm)

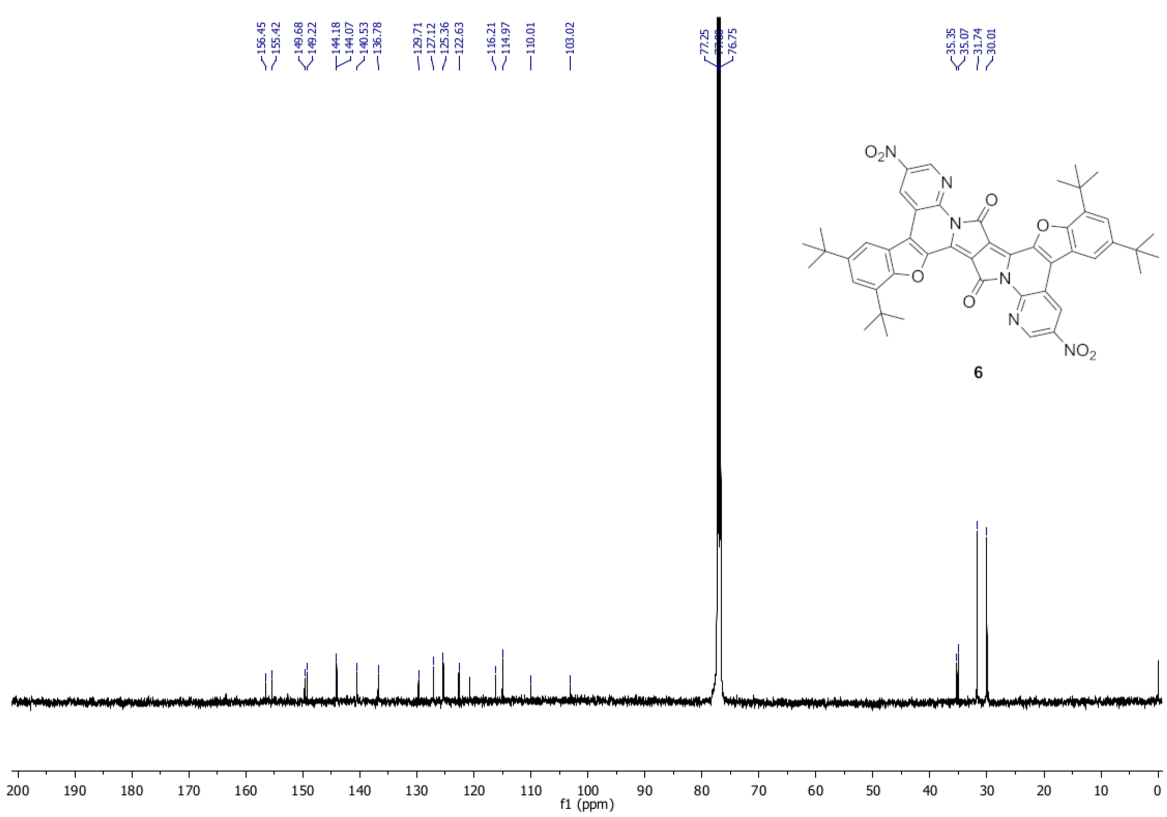
156.45
155.42
149.68
149.22
144.18
144.07
140.53
136.78
129.71
127.12
125.36
122.63
116.21
114.97
110.01
103.02
77.25
76.75
35.35
35.07
31.74
30.01
6
200 190 180 170 160 150 140 130 120 110 100 90 80 70 60 50 40 30 20 10 0
f1 (ppm)

4. Photophysical Data

4.1 Steady-State Absorption Spectroscopy

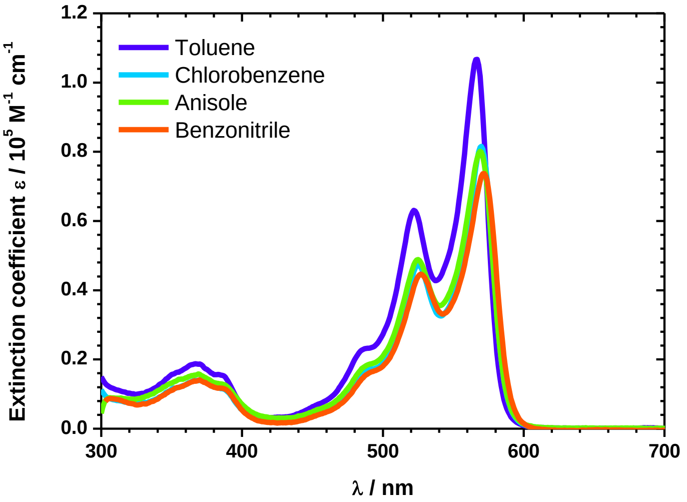

Figure S1. Absorption spectra of **3** in toluene, chlorobenzene, anisole and benzonitrile at room temperature.

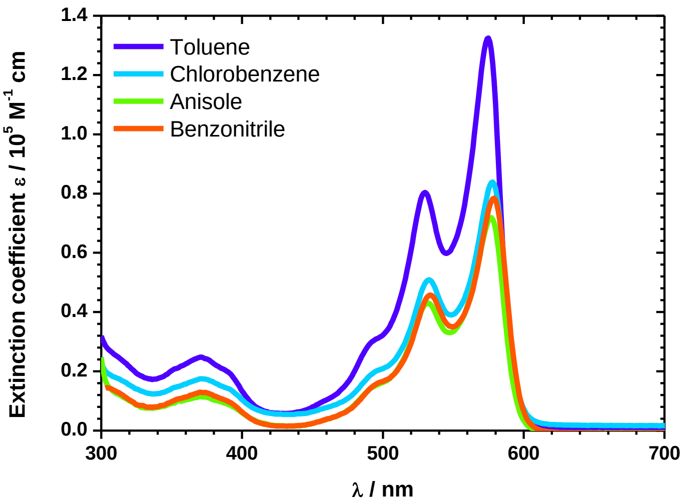

Figure S2. Absorption spectra of **4** in toluene, chlorobenzene, anisole and benzonitrile at room temperature.

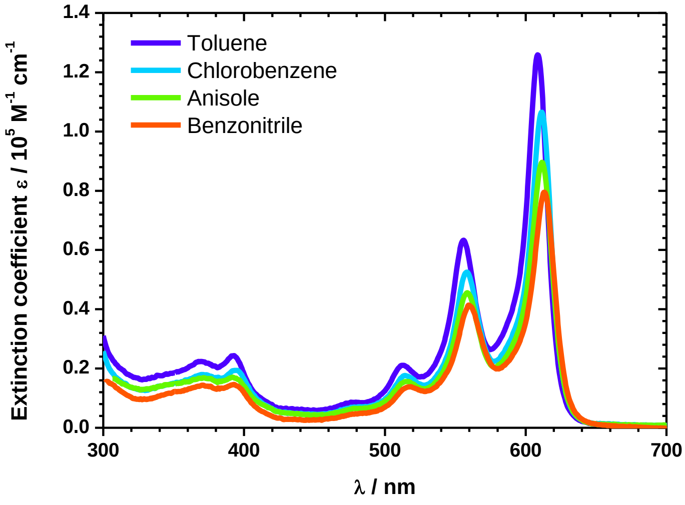

Figure S3. Absorption spectra of **5** in toluene, chlorobenzene, anisole and benzonitrile at room temperature.

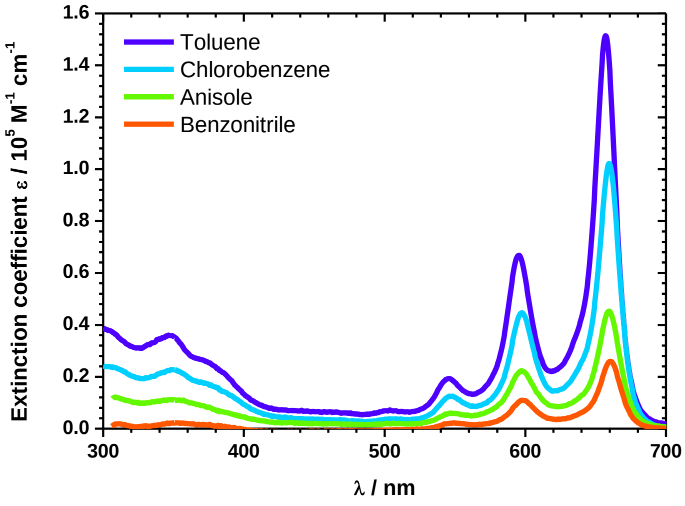

Figure S4. Absorption spectra of **6** in toluene, chlorobenzene, anisole and benzonitrile at room temperature.

Table S1: Maxima of absorption bands in toluene (top) and extinction coefficients in toluene, anisole, chlorobenzene, and benzonitrile (bottom).

	λ(S_{0-3}) / nm	λ(S_{0-2}) / nm	λ(S_{0-1}) / nm	λ(S_{0-0}) / nm
**3**	453	489	522	567
**4**	459	494	530	575
**5**	478	513	556	609
**6**	504	545	595	657

	Toluene	Anisole	Chlorobenzene	Benzonitrile
**3**	$10.8x\ 10^4$	$8.4 * 10^4$	$8.5 * 10^4$	$7.5 * 10^4$
**4**	$13.0 * 10^4$	$7.5 * 10^4$	$8.4 * 10^4$	$8.0 * 10^4$
**5**	$12.6 * 10^4$	$6.9 * 10^4$	$10.6 * 10^4$	$8.4 * 10^4$
**6**	$14.6 * 10^4$	$3.6 * 10^4$	$10.2 * 10^4$	$1.7 * 10^4$

Table S2: Maxima of fluorescence bands in toluene (top) and QYs (down) in toluene, anisole, chlorobenzene, and benzonitrile. The fluorescence references used for determining the quantum yields are Rhodamine B for **3** and **4** and Sulforhodamine 101 for **5** and **6**.

	λ(S_{0-0}) / nm	λ(S_{0-1}) / nm	λ(S_{0-2}) / nm
**3**	575	625	681
**4**	585	636	693
**5**	615	676	739
**6**	663	735	815

	Toluene	Anisole	Chlorobenzene	Benzonitrile
**3**	6.0%	0.6%	0.5%	0.4%
**4**	34.5%	0.5%	0.3%	0.2%
**5**	38.7%	1.8%	2.9%	0.6%
**6**	88.1%	58.3%	58.8%	3.2%

4.2 Steady-State Fluorescence Spectroscopy

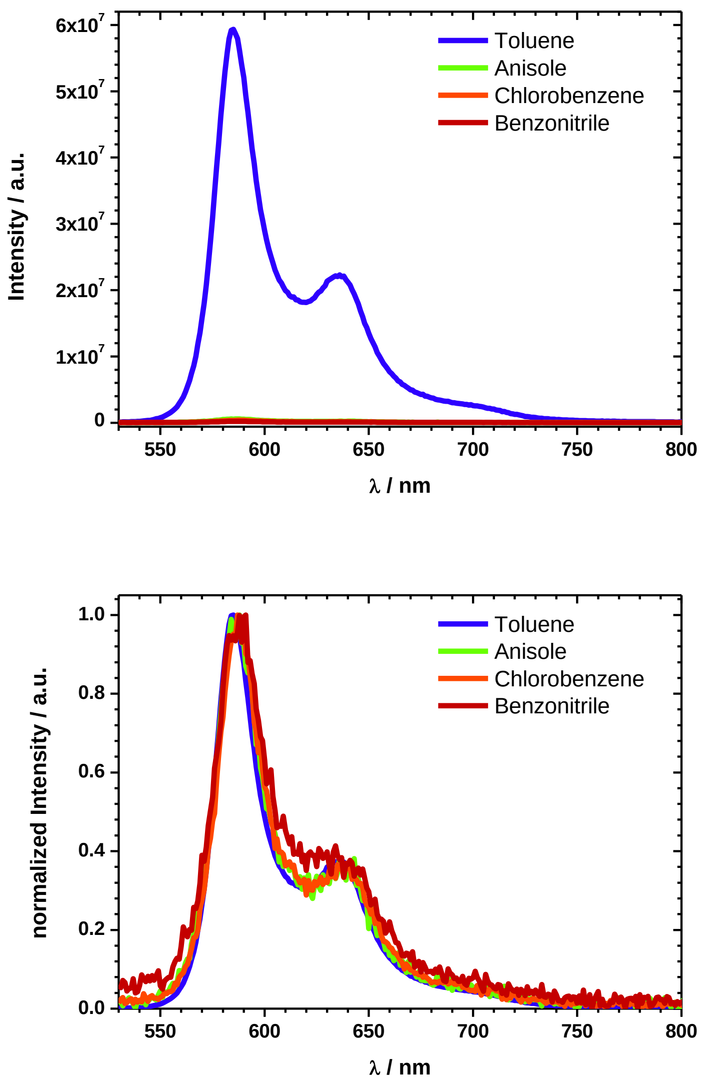

Figure S5. Steady-state fluorescence spectra (top) and normalized (bottom) of **3** (c = 3 x 10^{-6} M) in toluene, anisole, chlorobenzene and benzonitrile at room temperature. The excitation wavelength is 515 nm.

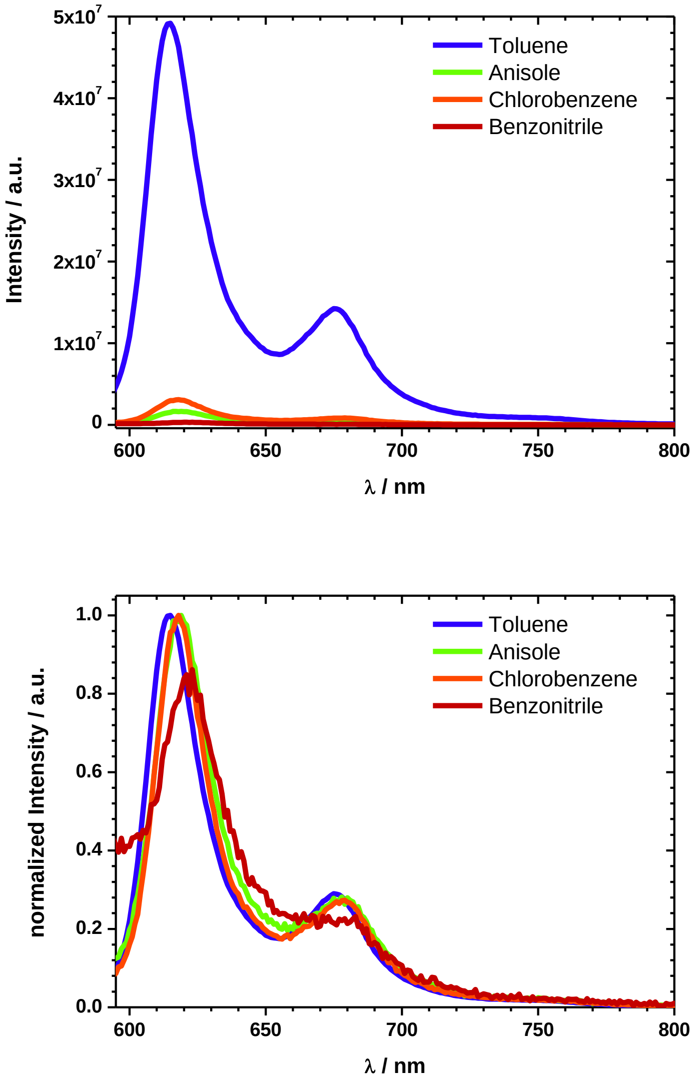

Figure S6. Steady-state fluorescence spectra (top) and normalized (bottom) of **4** (c = 3 x 10^{-6} M) in toluene, anisole, chlorobenzene and benzonitrile at room temperature. The excitation wavelength is 550 nm.

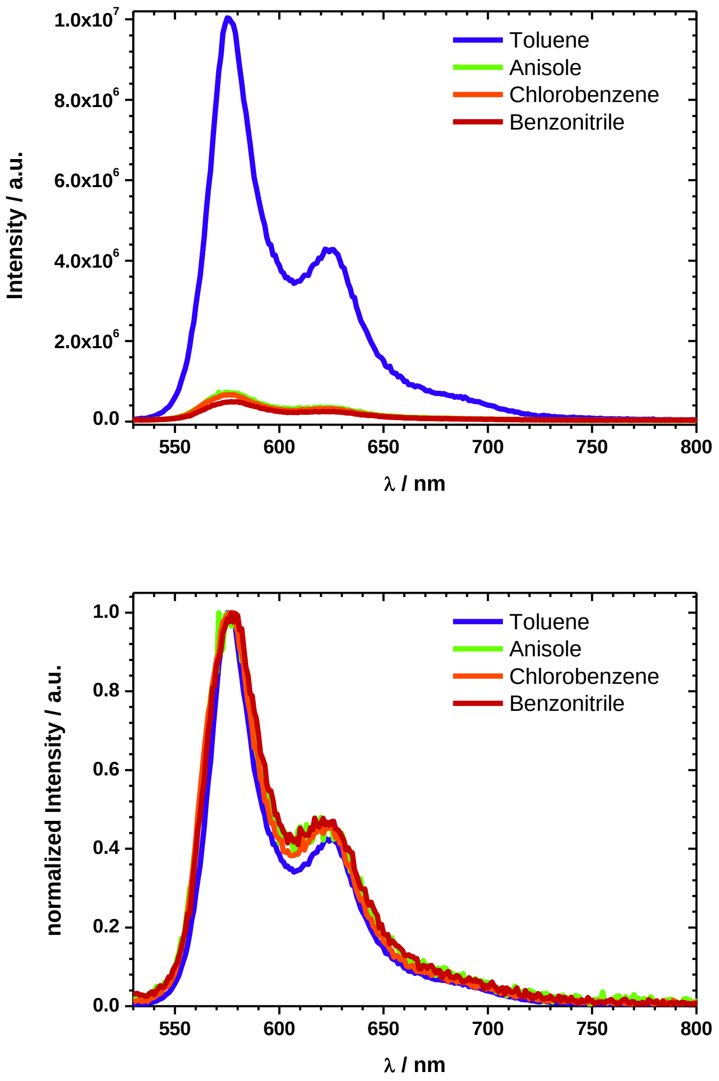

Figure S7. Steady-state fluorescence spectra (top) and normalized (bottom) of **5** (c = 3 x 10^{-6} M) in toluene, anisole, chlorobenzene and benzonitrile at room temperature. The excitation wavelength is 515 nm.

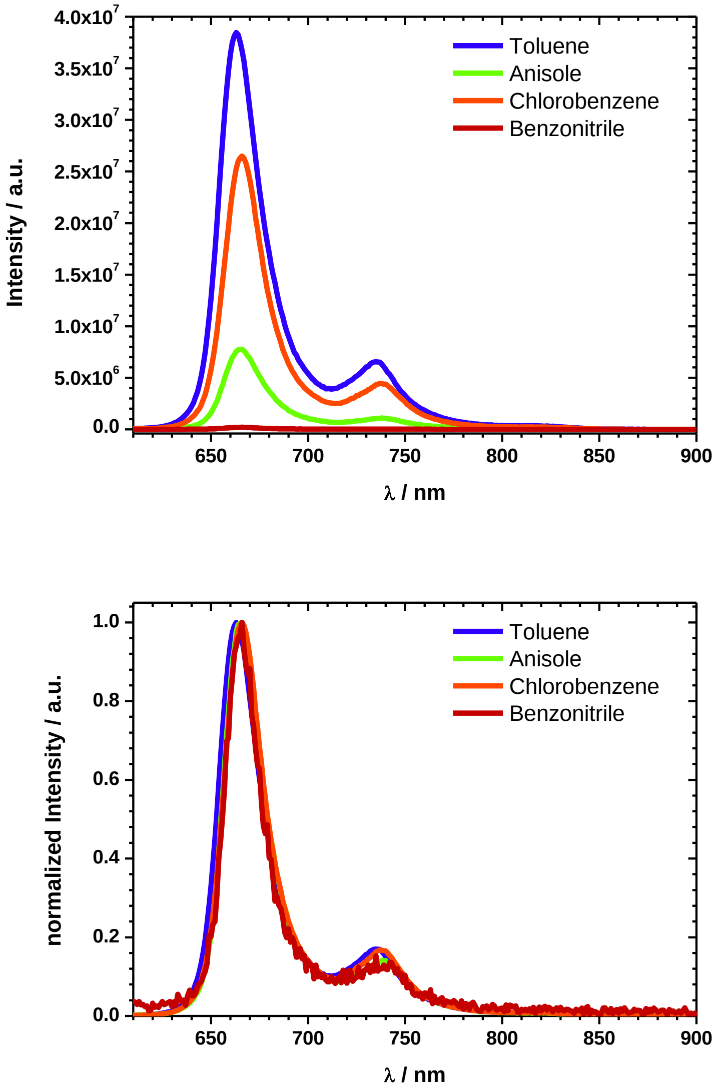

Figure S8. Steady-state fluorescence spectra (top) and normalized (bottom) of **6** (c = 3 x 10^{-6} M) in toluene, anisole, chlorobenzene and benzonitrile at room temperature. The excitation wavelength is 590 nm.

4.1 Electrochemistry

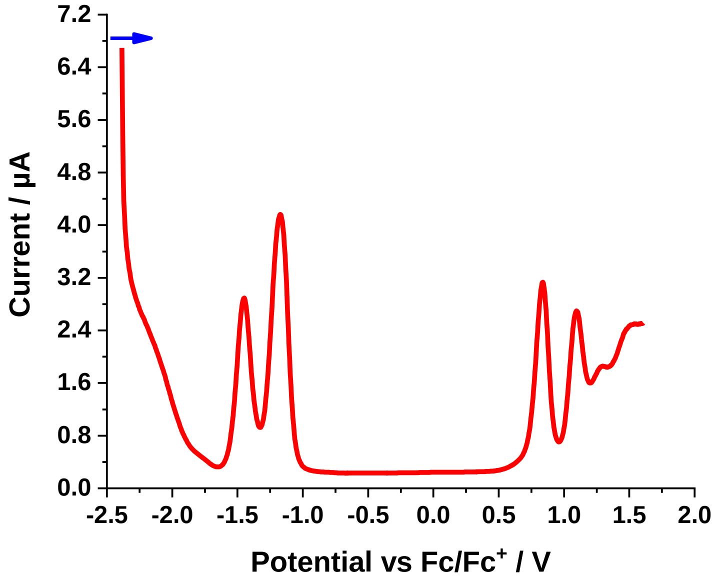

Figure S9. Differential pulse voltammogram of **4** in argon saturated dichloromethane (1.5 x 10^{-3} M) with 0.1 M $TBAPF_6$ at 0 °C. The blue arrow indicates the scan direction.

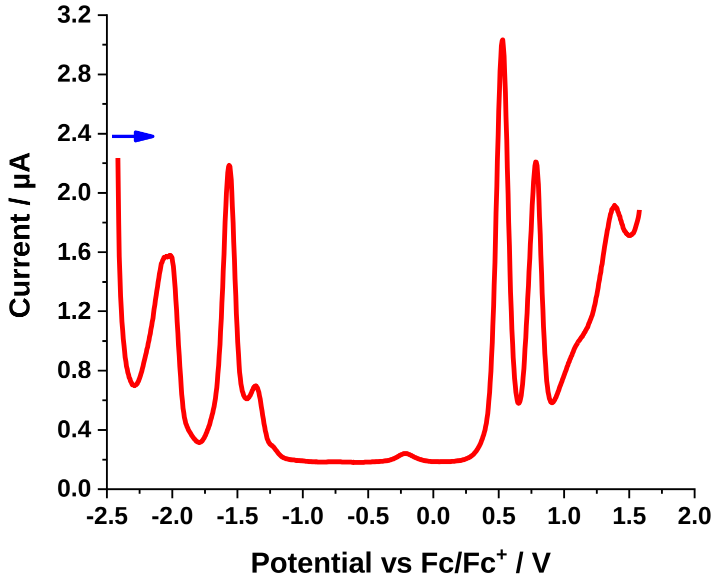

Figure S10. Differential pulse voltammogram of **5** in argon saturated dichloromethane (1.5 x 10^{-3} M) with 0.1 M $TBAPF_6$ at 0 °C. The blue arrow indicates the scan direction.

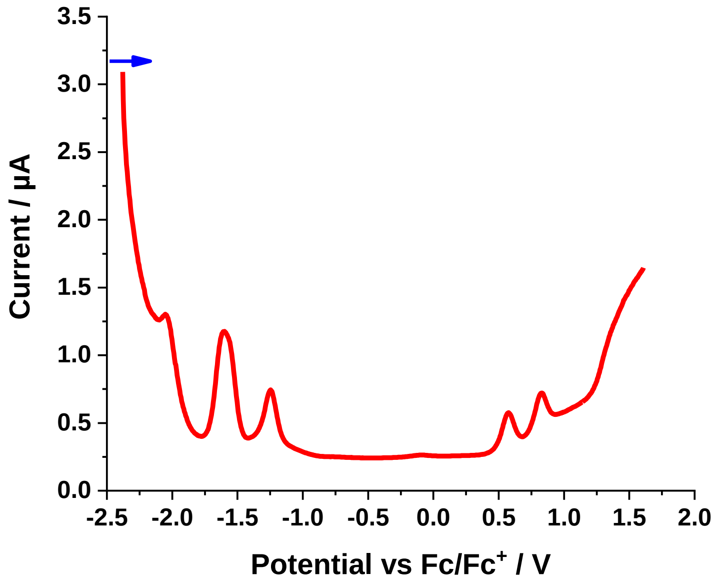

Figure S11. Differential pulse voltammogram of **6** in argon saturated dichloromethane (1.5 x 10^{-3} M) with 0.1 M $TBAPF_6$ at 0 °C. The blue arrow indicates the scan direction.

4.2 Spectroelectrochemistry

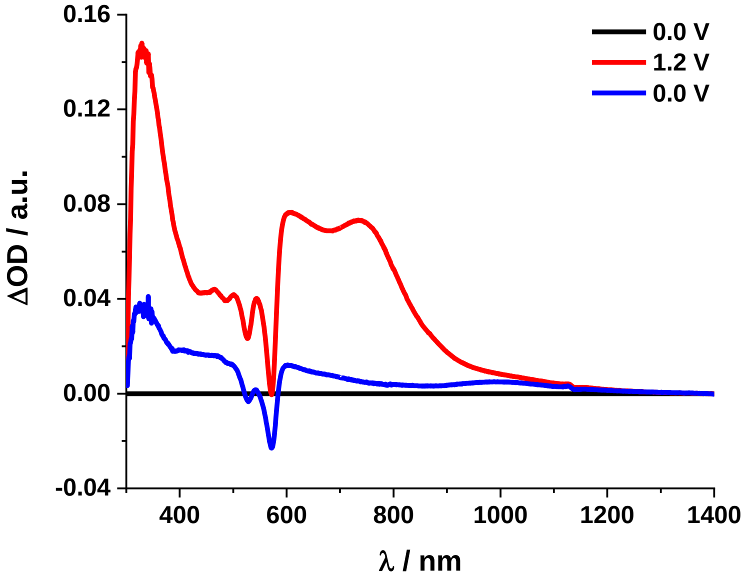

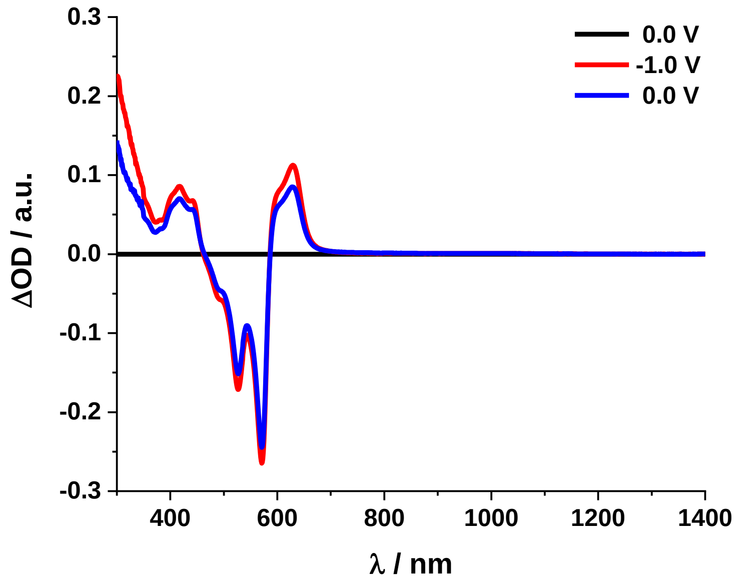

Figure S12. Differential absorption spectra of **3** upon applying an oxidative (top, red) and reductive (bottom, red) potential, respectively. Spectra were taken from an argon saturated benzonitrile solution containing 0.1 M $TBAPF_6$ as supporting electrolyte. The applied potential was neutralized to check for reversibility (blue).

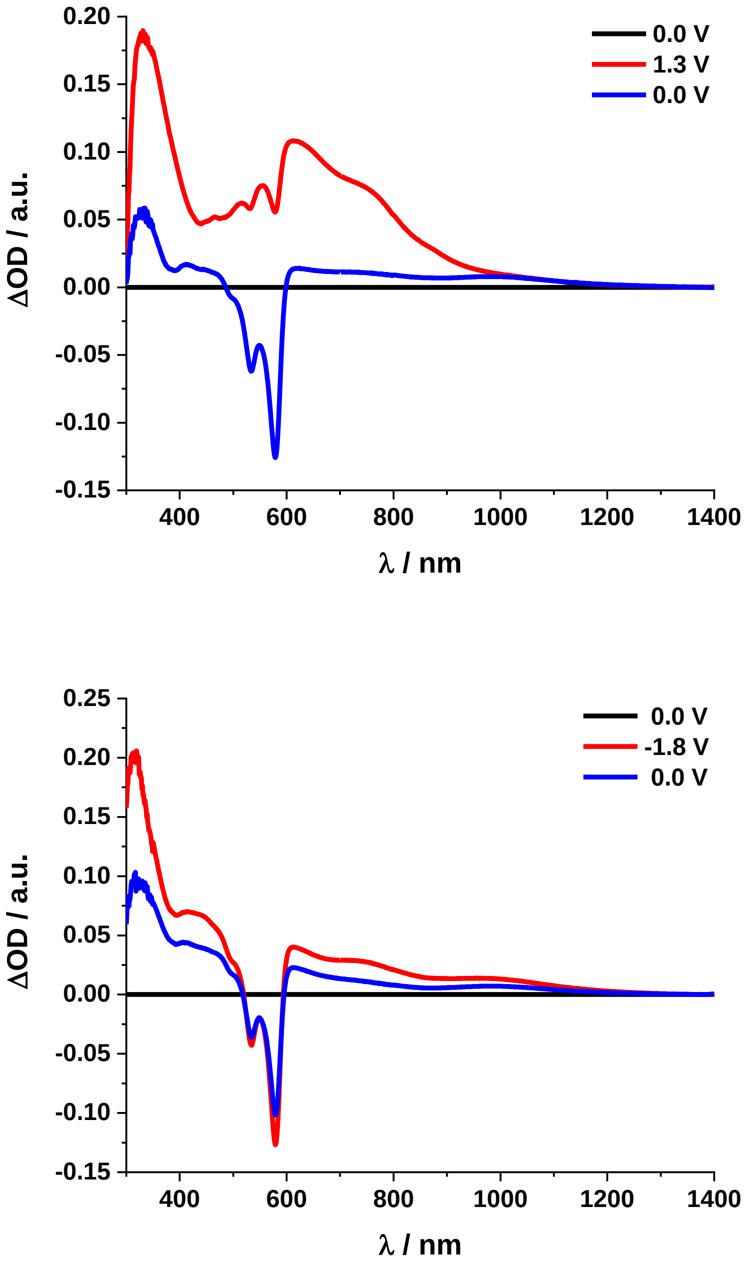

Figure S13. Differential absorption spectra of **4** upon applying an oxidative (top, red) and reductive (bottom, red) potential, respectively. Spectra were taken from an argon saturated benzonitrile solution containing 0.1 M $TBAPF_6$ as supporting electrolyte. The applied potential was neutralized to check for reversibility (blue).

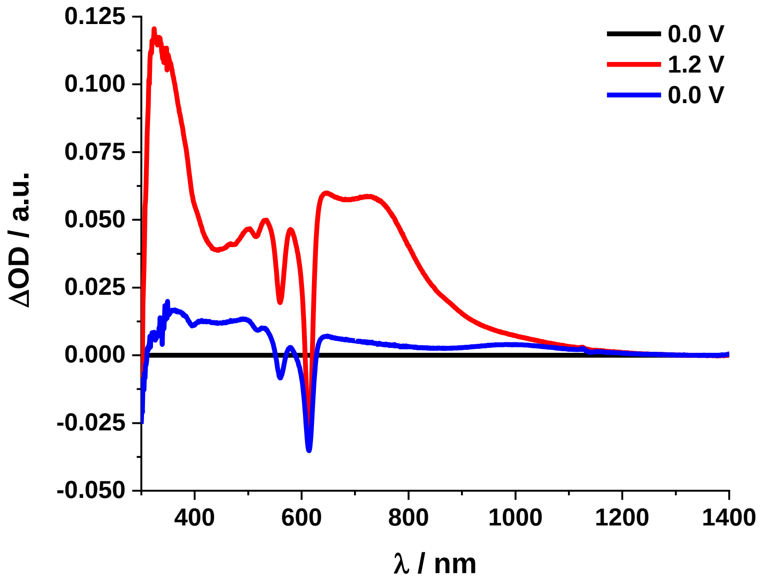

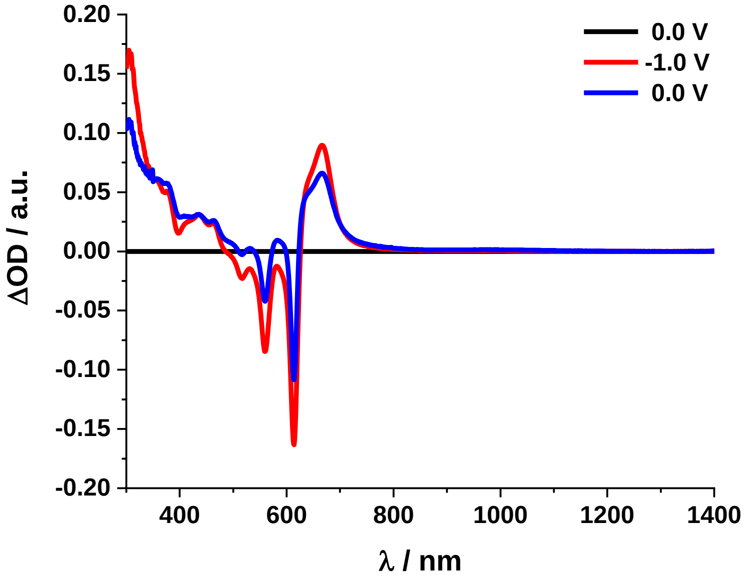

Figure S14. Differential absorption spectra of **5** upon applying an oxidative (top, red) and reductive (bottom, red) potential, respectively. Spectra were taken from an argon saturated benzonitrile solution containing 0.1 M $TBAPF_6$ as supporting electrolyte. The applied potential was neutralized to check for reversibility (blue).

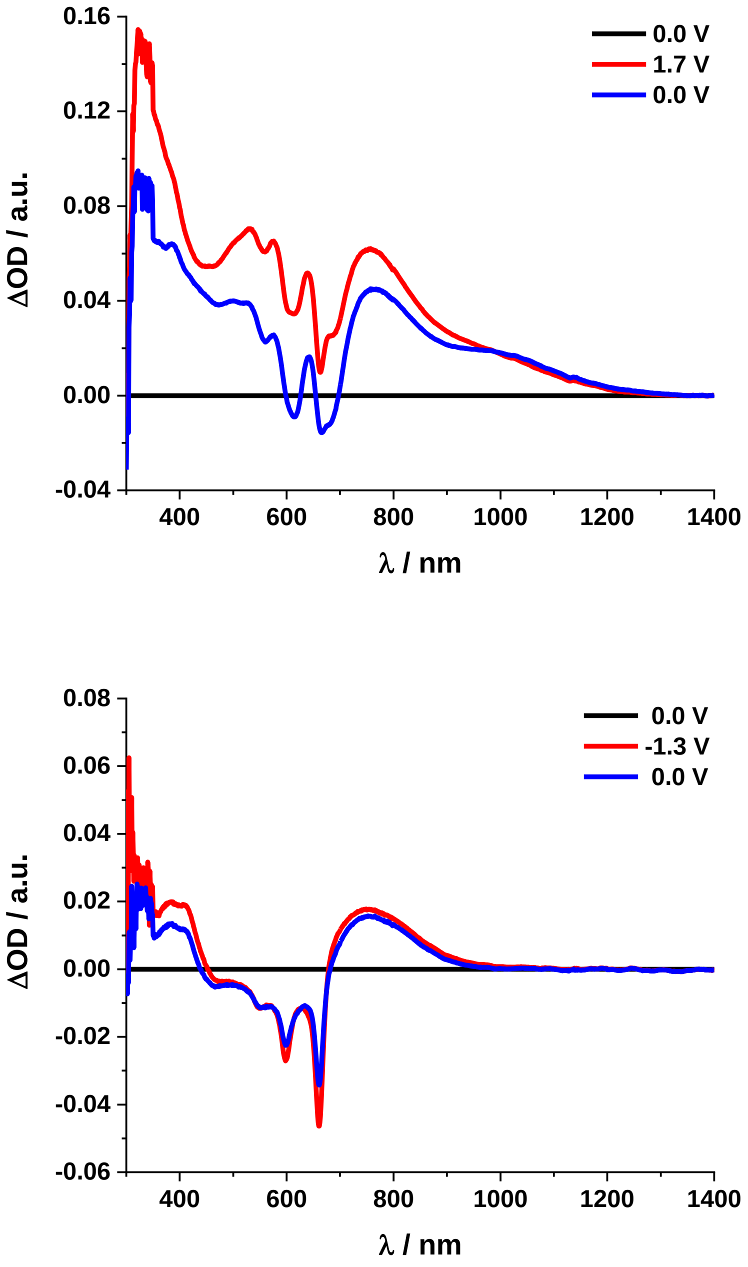

Figure S15. Differential absorption spectra of **6** upon applying an oxidative (top, red) and reductive (bottom, red) potential, respectively. Spectra were taken from an argon saturated benzonitrile solution containing 0.1 M $TBAPF_6$ as supporting electrolyte. The applied potential was neutralized to check for reversibility (blue).

4.3 Transient Absorption Spectroscopy

In Toluene

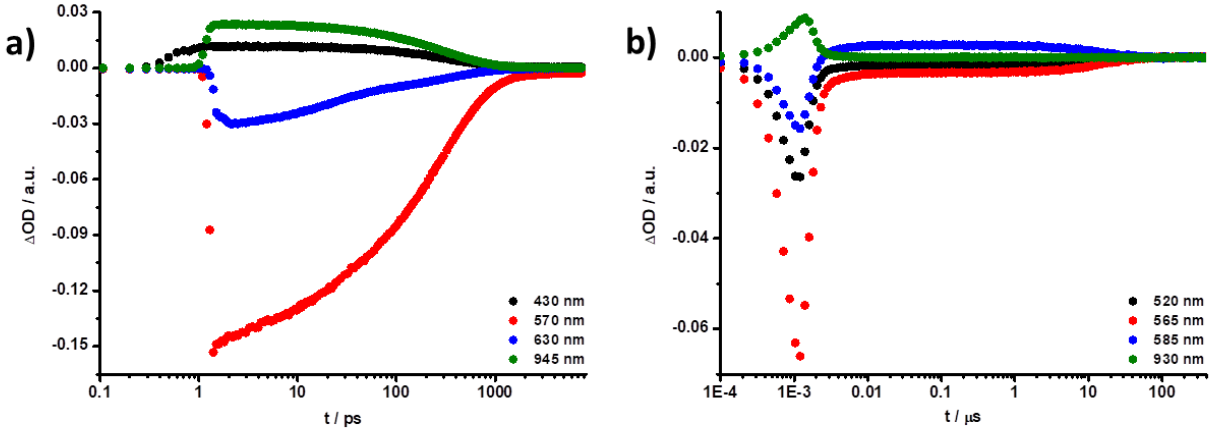

Figure S16. a) Kinetics obtained from multiwavelength analysis at the respective wavelengths from fsTA experiments (550 nm) of **3** in toluene (Figure 9). b) Kinetics obtained from multiwavelength analysis at the respective wavelengths from nsTA experiments (550 nm) of **3** in toluene (Figure 10).

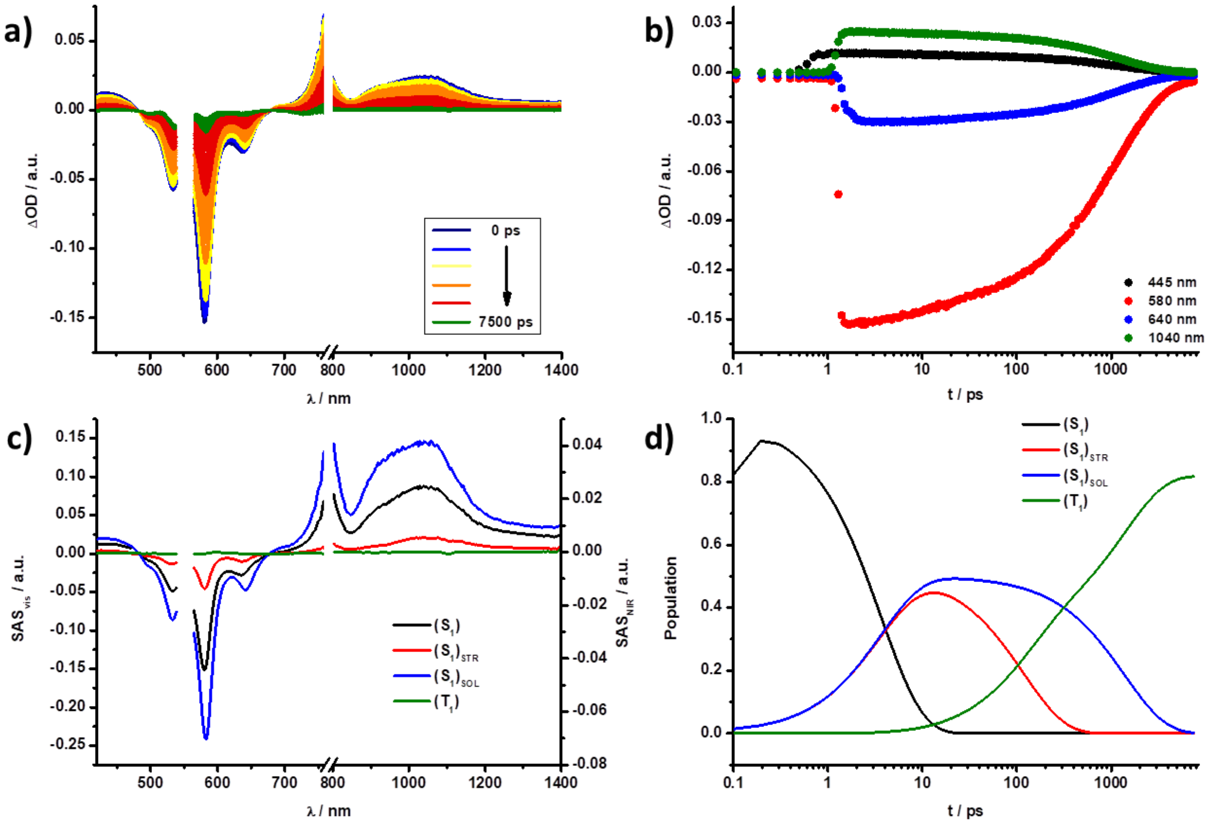

Figure S17. (a) – Differential absorption spectra obtained upon fsTA experiments (550 nm) of **4** in toluene with several time delays between 0 and 7500 ps at rt. (b) – Kinetics obtained from multi-wavelength analysis at the respective wavelengths (c) – Species associated spectra of the transient absorption data of **4** shown in a), with the initially formed singlet excited state (S_1) (black), structurally relaxed singlet excited state $(S_1)_{STR}$ (red), solvent reorganized singlet excited state $(S_1)_{SOL}$ (blue), and the triplet excited state (T_1) (green) obtained from GloTarAn target analysis. (d) – Relative population kinetics of the respective states.

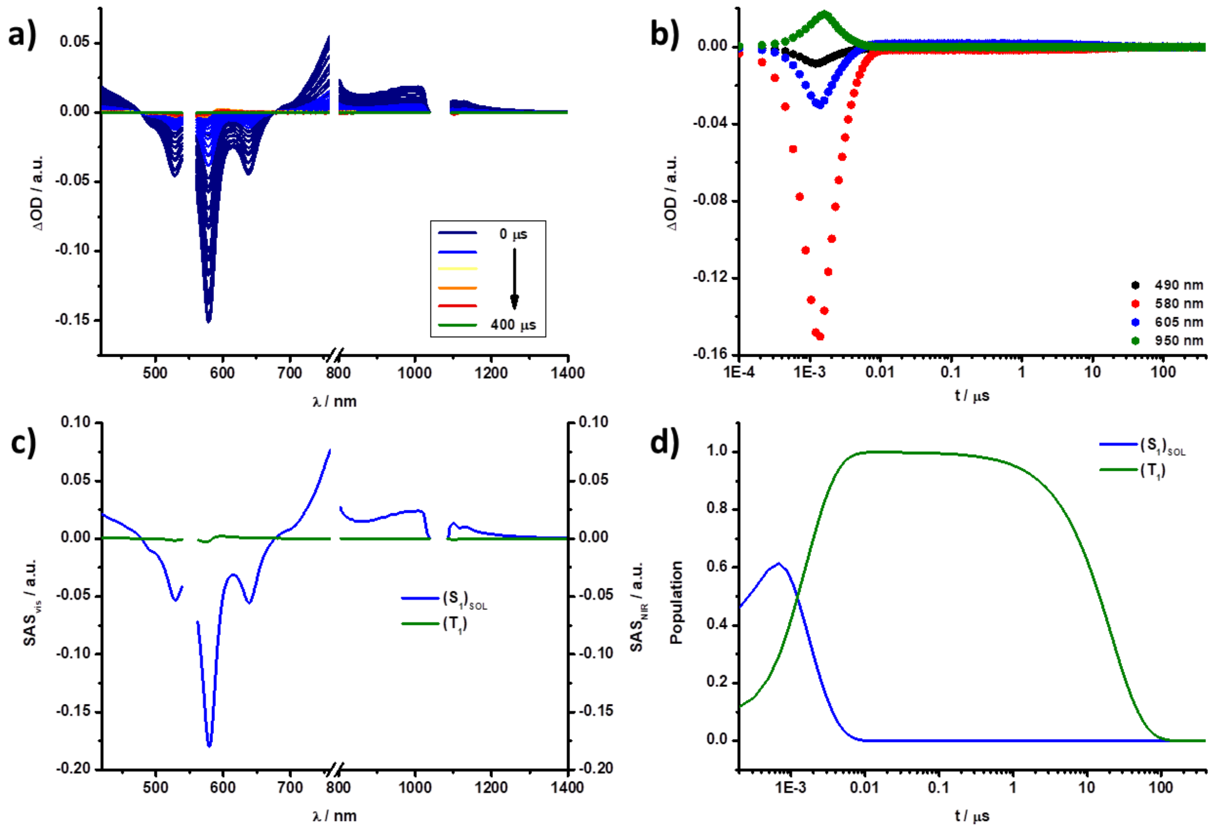

Figure S18. (a) – Differential absorption spectra obtained upon nsTA experiments (550 nm) of **4** in toluene with several time delays between 0 and 400 μs at rt. (b) – Kinetics obtained from multi-wavelength analysis at the respective wavelengths. (c) – Species associated spectra of the transient absorption data of **4** shown in a), with the solvent reorganized singlet excited state $(S_1)_{SOL}$ (blue) and the triplet excited state (T_1) (green) obtained from GloTarAn target analysis. (d) – Relative population kinetics of the respective states.

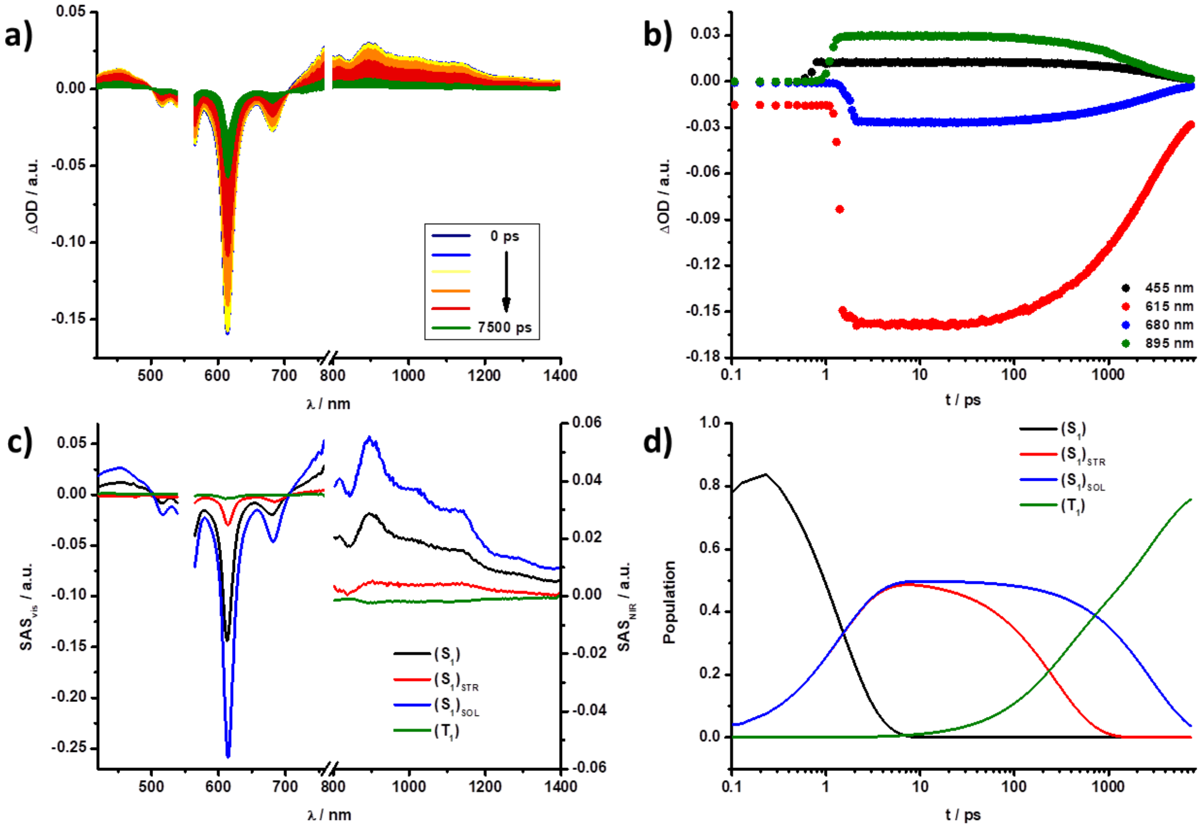

Figure S19. (a) – Differential absorption spectra obtained upon fsTA experiments (550 nm) of **5** in toluene with several time delays between 0 and 7500 ps at rt. (b) – Kinetics obtained from multi-wavelength analysis at the respective wavelengths (c) – Species associated spectra of the transient absorption data of **5** shown in a), with the initially formed singlet excited state (S_1) (black), structurally relaxed singlet excited state $(S_1)_{STR}$ (red), solvent reorganized singlet excited state $(S_1)_{SOL}$ (blue), and the triplet excited state (T_1) (green) obtained from GloTarAn target analysis. (d) – Relative population kinetics of the respective states.

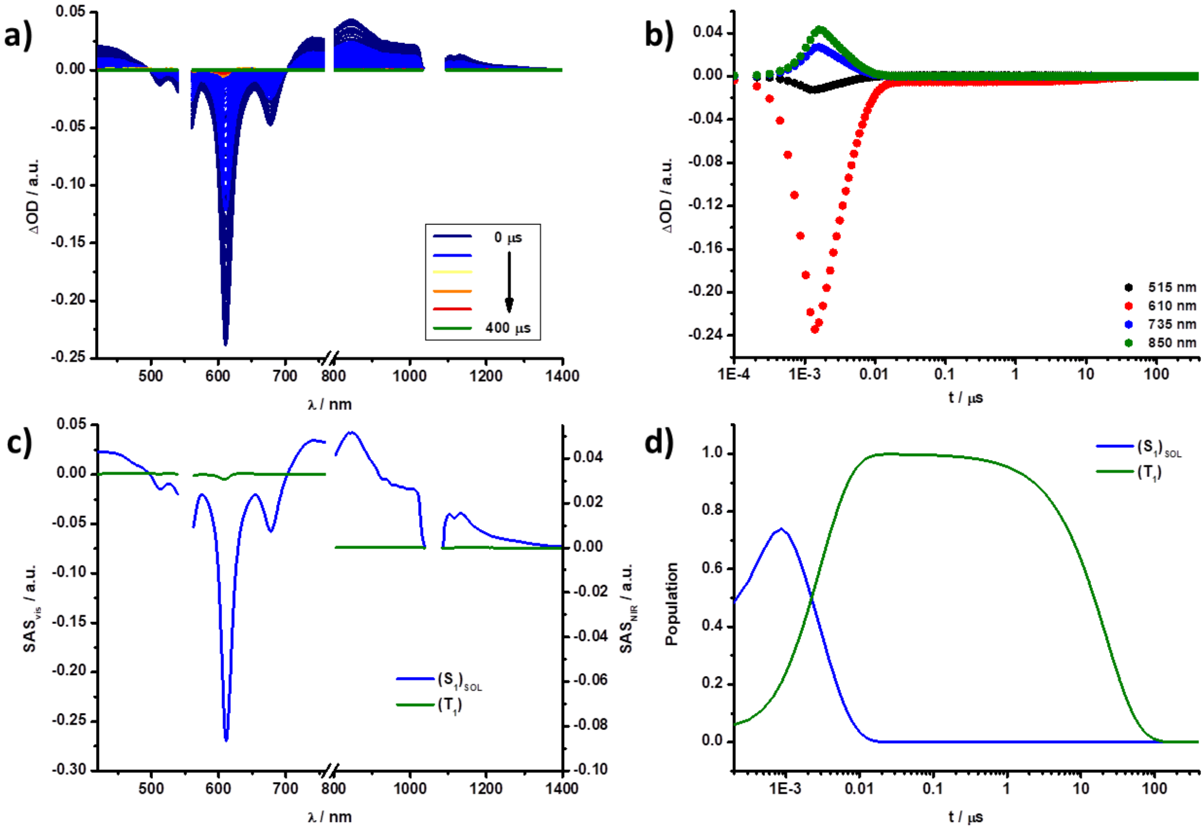

Figure S20. (a) – Differential absorption spectra obtained upon nsTA experiments (550 nm) of **5** in toluene with several time delays between 0 and 400 µs at rt. (b) – Kinetics obtained from multi-wavelength analysis at the respective wavelengths. (c) – Species associated spectra of the transient absorption data of **5** shown in a), with the solvent reorganized singlet excited state $(S_1)_{SOL}$ (blue) and the triplet excited state (T_1) (green) obtained from GloTarAn target analysis. (d) – Relative population kinetics of the respective states.

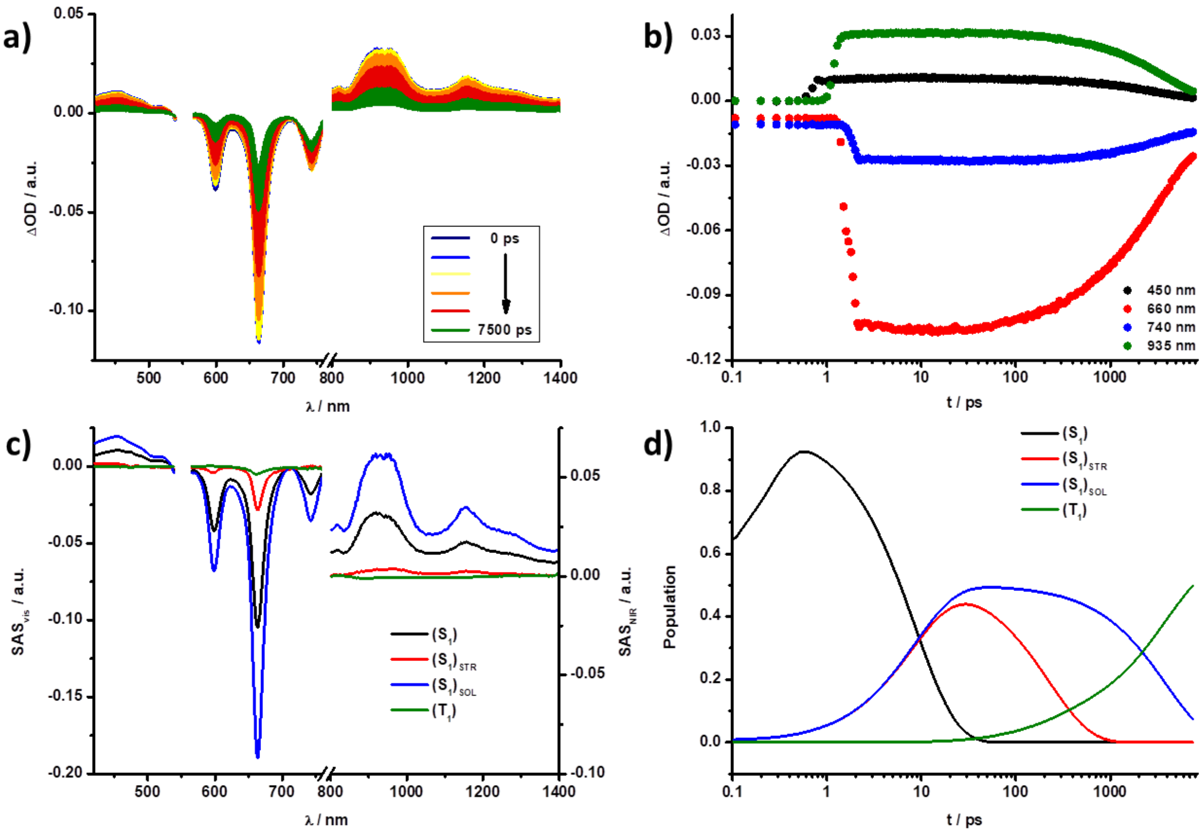

Figure S21. (a) – Differential absorption spectra obtained upon fsTA experiments (550 nm) of **6** in toluene with several time delays between 0 and 7500 ps at rt. (b) – Kinetics obtained from multi-wavelength analysis at the respective wavelengths (c) – Species associated spectra of the transient absorption data of **6** shown in a), with the initially formed singlet excited state (S_1) (black), structurally relaxed singlet excited state $(S_1)_{STR}$ (red), solvent reorganized singlet excited state $(S_1)_{SOL}$ (blue), and the triplet excited state (T_1) (green) obtained from GloTarAn target analysis. (d) – Relative population kinetics of the respective states.

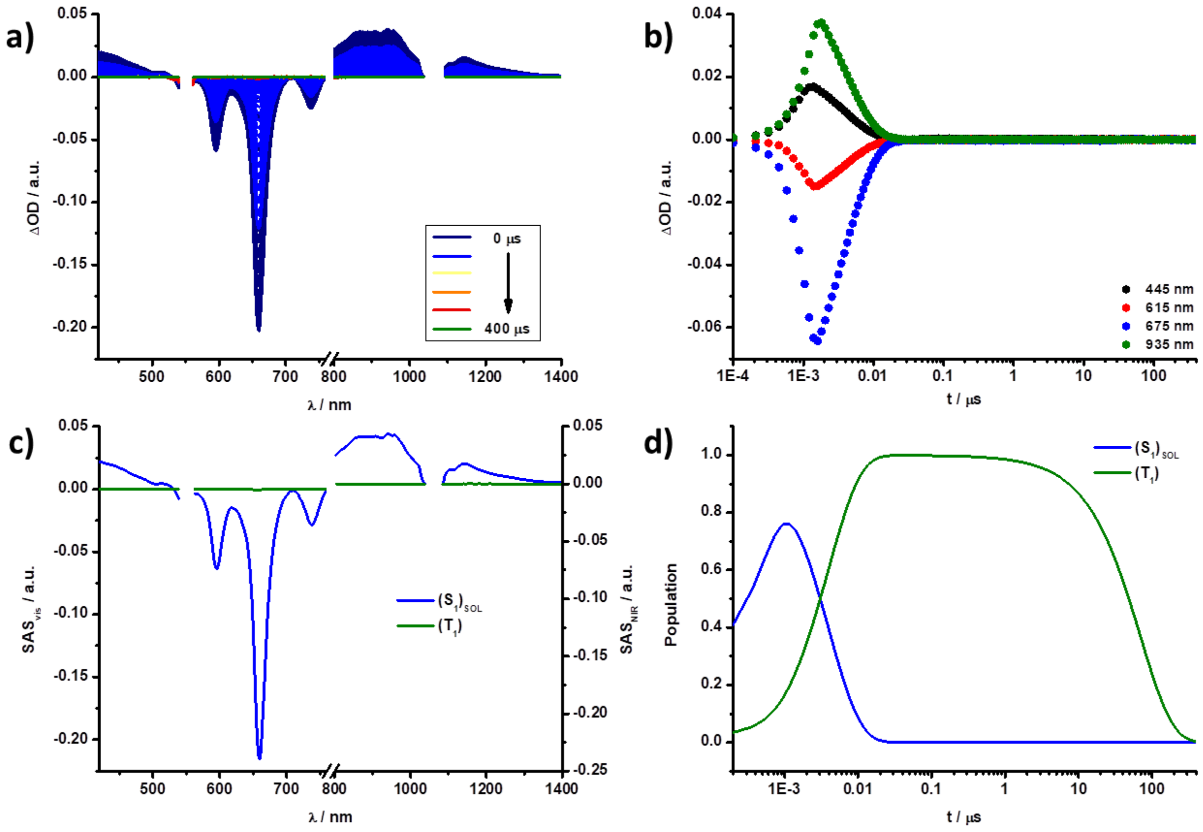

Figure S22. (a) – Differential absorption spectra obtained upon nsTA experiments (550 nm) of **6** in toluene with several time delays between 0 and 400 µs at rt. (b) – Kinetics obtained from multi-wavelength analysis at the respective wavelengths. (c) – Species associated spectra of the transient absorption data of **6** shown in a), with the solvent reorganized singlet excited state $(S_1)_{SOL}$ (blue) and the triplet excited state (T_1) (green) obtained from GloTarAn target analysis. (d) – Relative population kinetics of the respective states.

In Benzonitrile

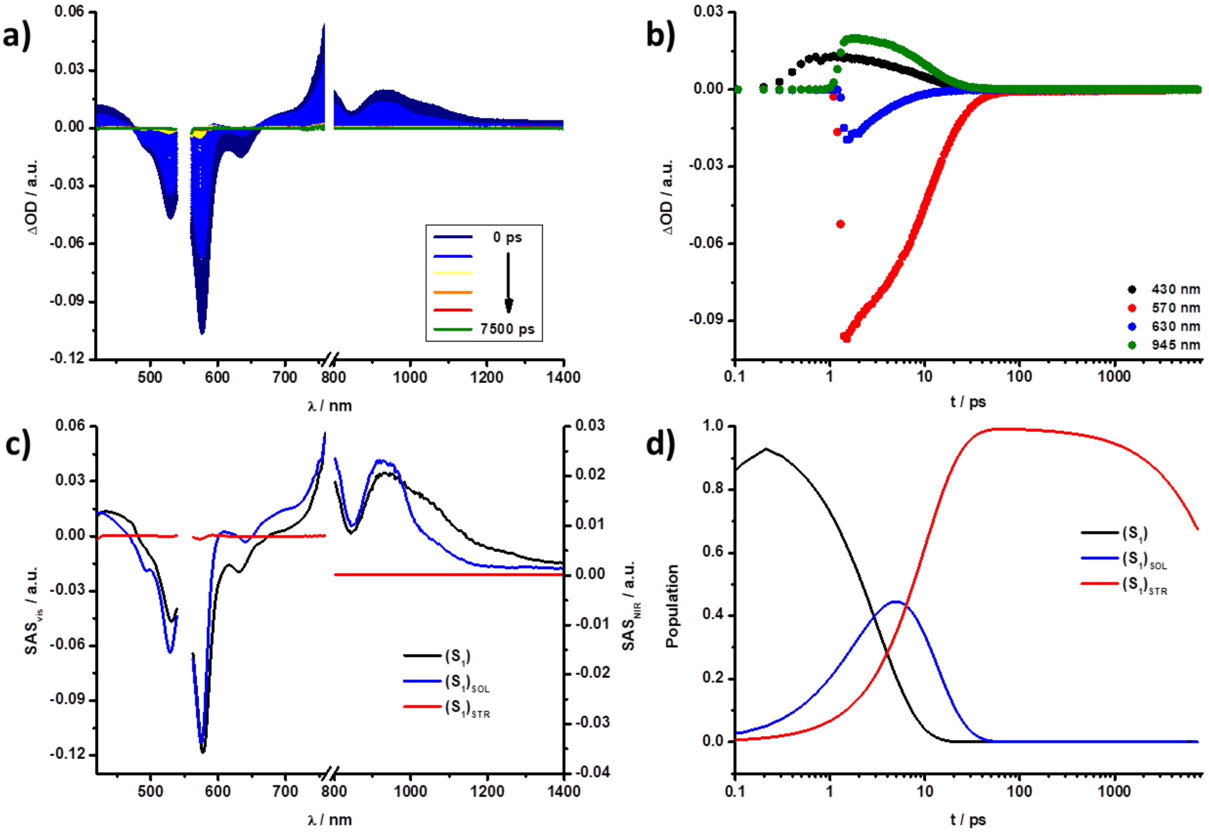

Figure S23. (a) – Differential absorption spectra obtained upon fsTA experiments (550 nm) of **3** in benzonitrile with several time delays between 0 and 7500 ps at rt. (b) – Kinetics obtained from multi-wavelength analysis at the respective wavelengths (c) – Species associated spectra of the transient absorption data of **3** shown in a), with the initially formed singlet excited state (S_1) (black), solvent reorganized singlet excited state $(S_1)_{SOL}$ (blue), and structurally relaxed singlet excited state $(S_1)_{STR}$ (red) obtained from GloTarAn target analysis. (d) – Relative population kinetics of the respective states.

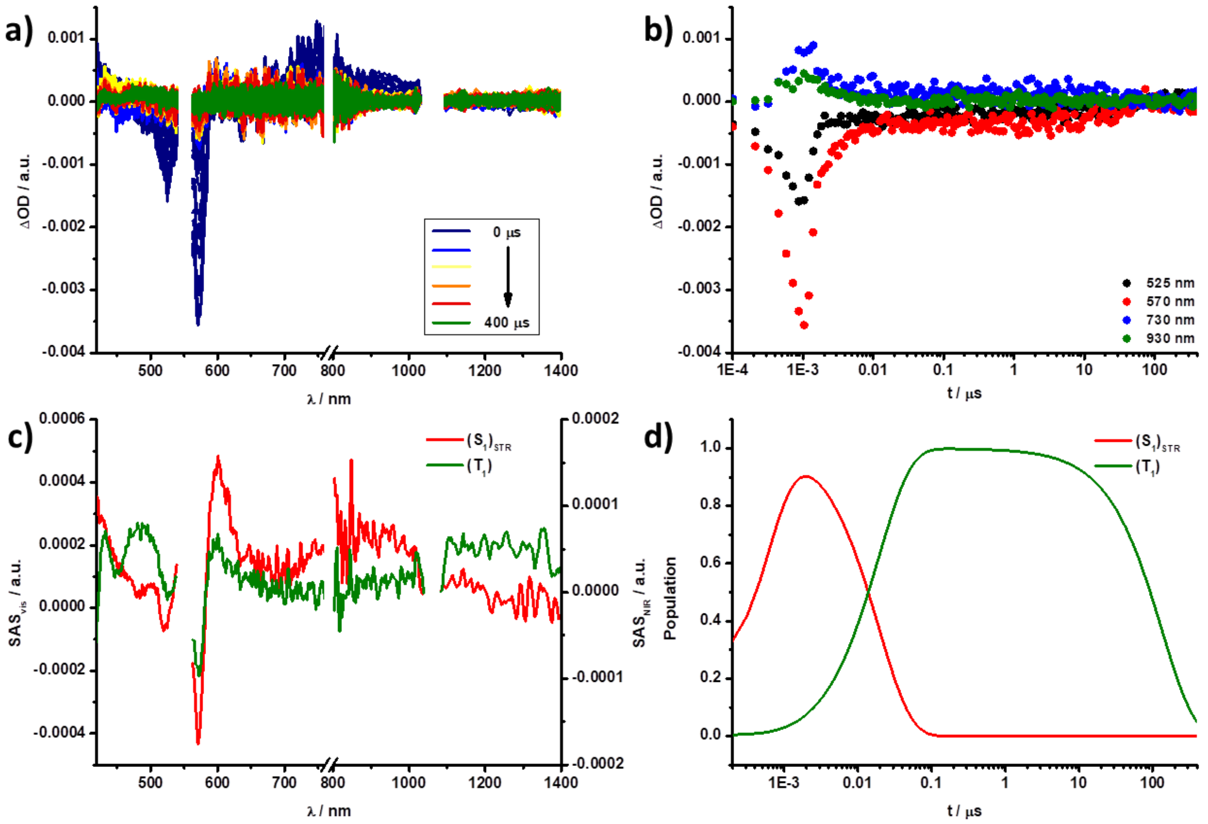

Figure S24. (a) – Differential absorption spectra obtained upon nsTA experiments (550 nm) of **3** in benzonitrile with several time delays between 0 and 400 μs at rt. (b) – Kinetics obtained from multi-wavelength analysis at the respective wavelengths. (c) – Species associated spectra of the transient absorption data of **3** shown in a), with the structurally relaxed singlet excited state $(S_1)_{STR}$ (red) and the triplet excited state (T_1) (green) obtained from GloTarAn target analysis. (d) – Relative population kinetics of the respective states.

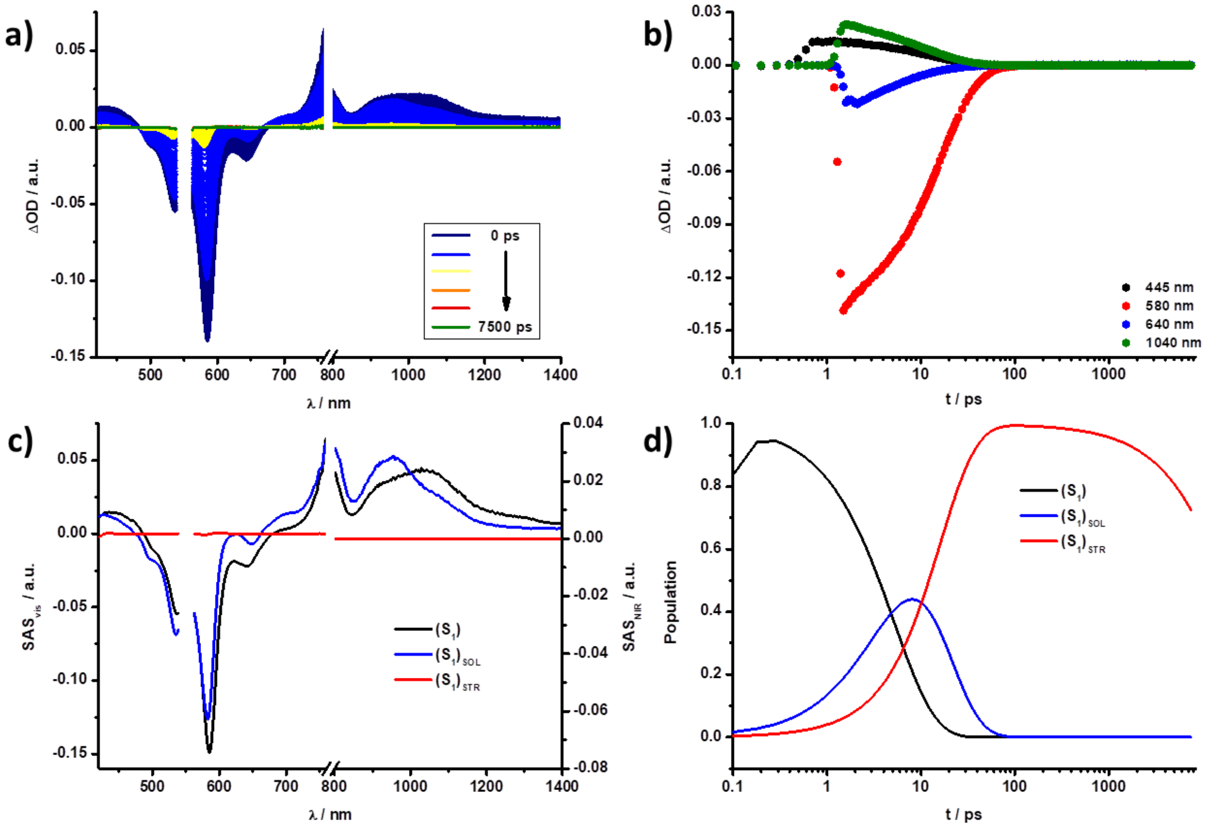

Figure S25. (a) – Differential absorption spectra obtained upon fsTA experiments (550 nm) of **4** in benzonitrile with several time delays between 0 and 7500 ps at rt. (b) – Kinetics obtained from multi-wavelength analysis at the respective wavelengths (c) – Species associated spectra of the transient absorption data of **4** shown in a), with the initially formed singlet excited state (S_1) (black), solvent reorganized singlet excited state $(S_1)_{SOL}$ (blue), and structurally relaxed singlet excited state $(S_1)_{STR}$ (red) obtained from GloTarAn target analysis. (d) – Relative population kinetics of the respective states.

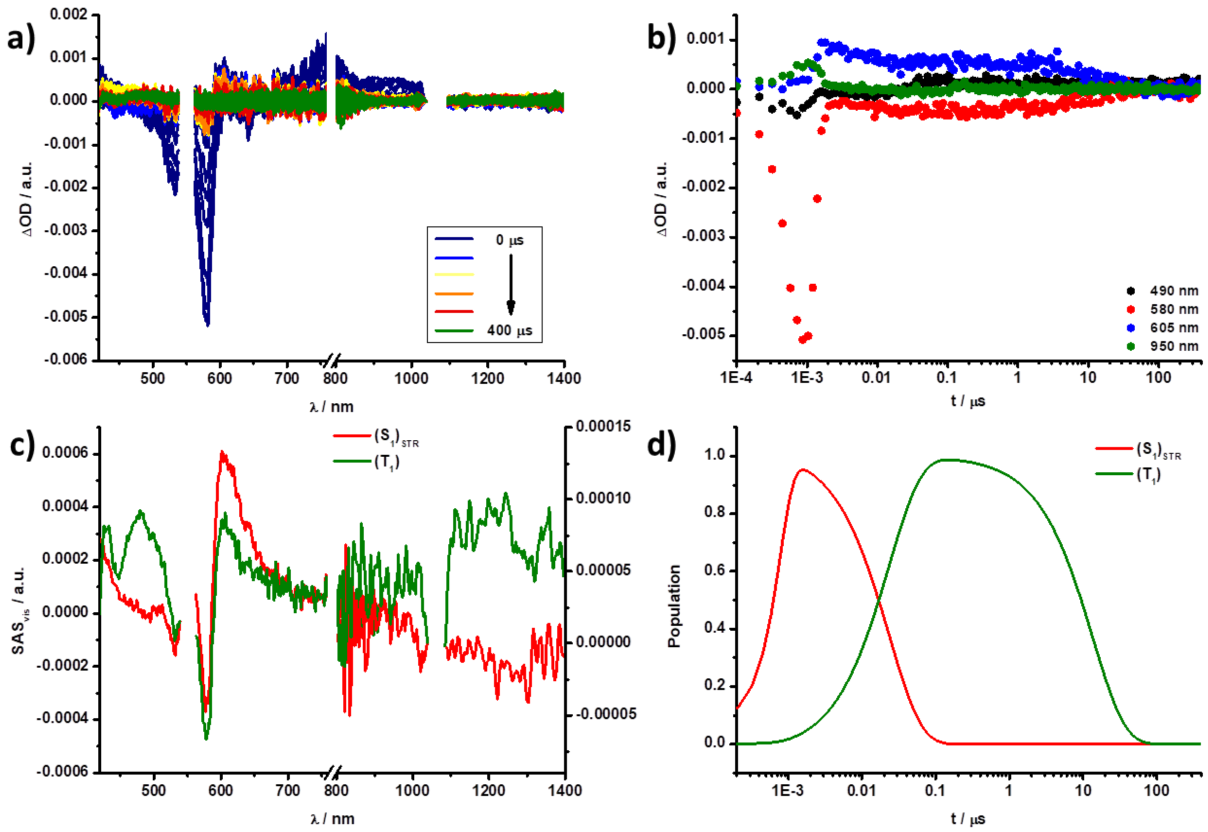

Figure S26. (a) – Differential absorption spectra obtained upon nsTA experiments (550 nm) of **4** in benzonitrile with several time delays between 0 and 400 µs at rt. (b) – Kinetics obtained from multi-wavelength analysis at the respective wavelengths. (c) – Species associated spectra of the transient absorption data of **4** shown in a), with the structurally relaxed singlet excited state $(S_1)_{STR}$ (red) and the triplet excited state (T_1) (green) obtained from GloTarAn target analysis. (d) – Relative population kinetics of the respective states.

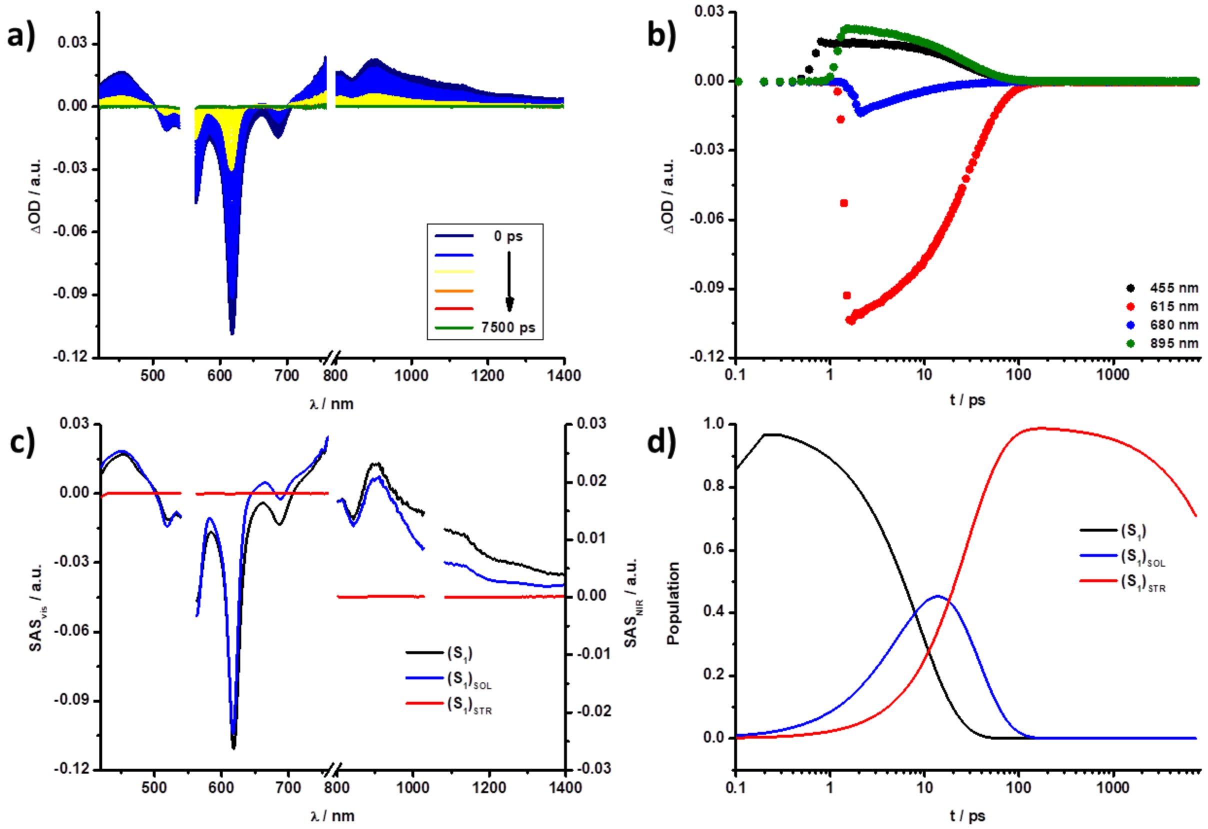

Figure S27. (a) – Differential absorption spectra obtained upon fsTA experiments (550 nm) of **5** in benzonitrile with several time delays between 0 and 7500 ps at rt. (b) – Kinetics obtained from multi-wavelength analysis at the respective wavelengths (c) – Species associated spectra of the transient absorption data of **5** shown in a), with the initially formed singlet excited state (S_1) (black), solvent reorganized singlet excited state $(S_1)_{SOL}$ (blue), and structurally relaxed singlet excited state $(S_1)_{STR}$ (red) (red) obtained from GloTarAn target analysis. (d) – Relative population kinetics of the respective states.

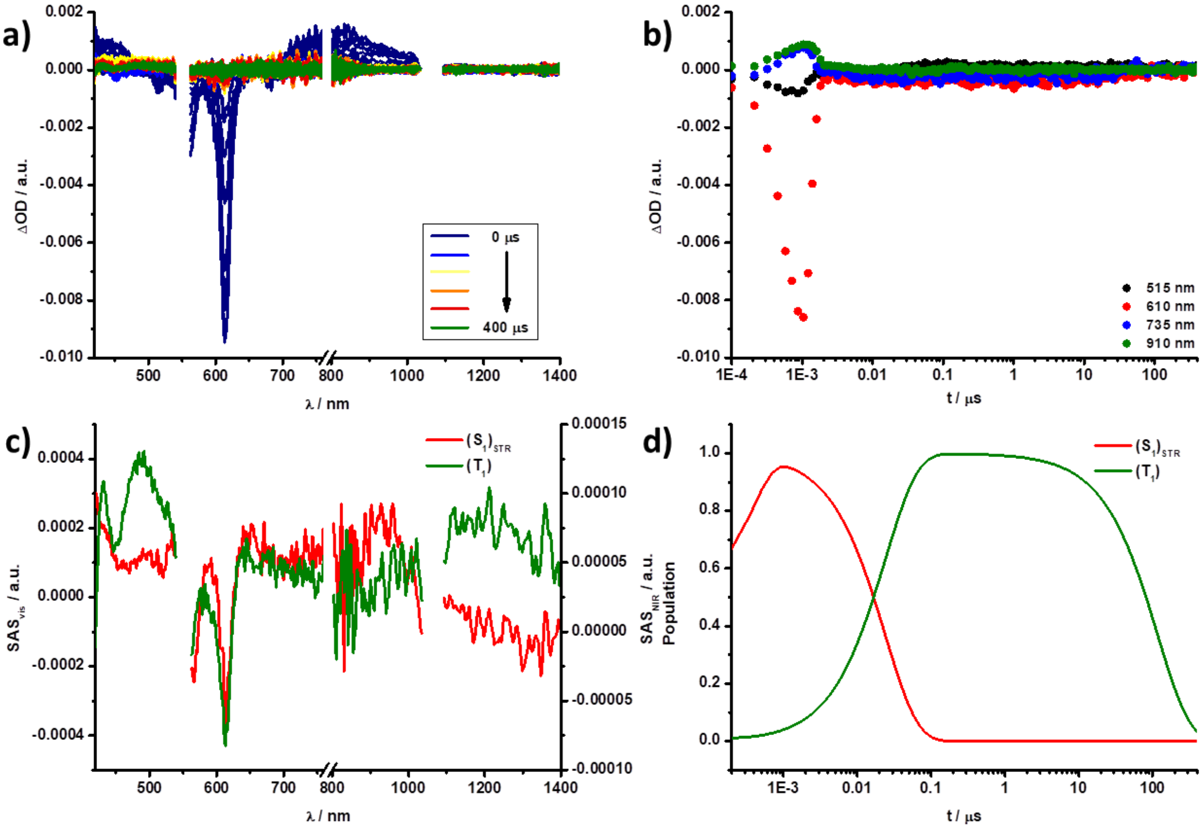

Figure S28. (a) – Differential absorption spectra obtained upon nsTA experiments (550 nm) of **5** in benzonitrile with several time delays between 0 and 400 µs at rt. (b) – Kinetics obtained from multi-wavelength analysis at the respective wavelengths. (c) – Species associated spectra of the transient absorption data of **5** shown in a), with the structurally relaxed singlet excited state $(S_1)_{STR}$ (red) and the triplet excited state (T_1) (green) obtained from GloTarAn target analysis. (d) – Relative population kinetics of the respective states.

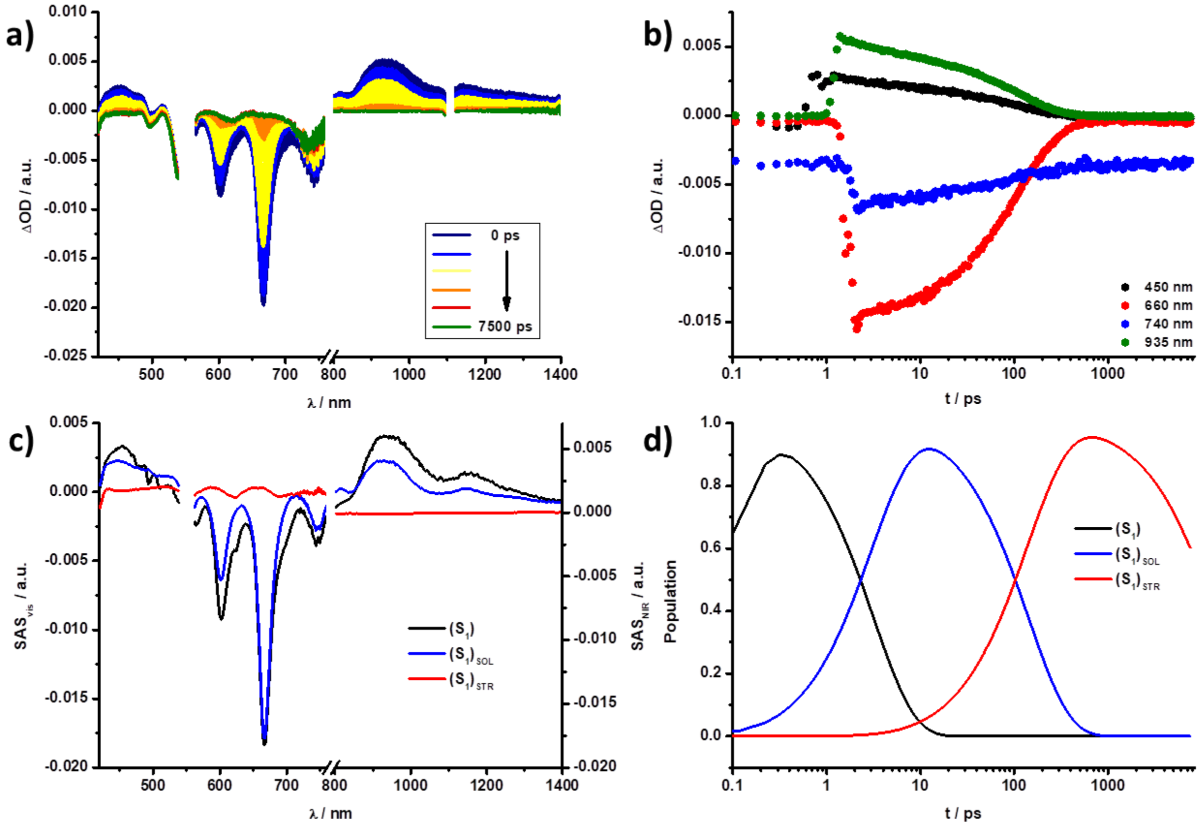

Figure S29. (a) – Differential absorption spectra obtained upon fsTA experiments (550 nm) of **6** in benzonitrile with several time delays between 0 and 7500 ps at rt. (b) – Kinetics obtained from multi-wavelength analysis at the respective wavelengths (c) – Species associated spectra of the transient absorption data of **6** shown in a), with the initially formed singlet excited state (S_1) (black), solvent reorganized singlet excited state $(S_1)_{SOL}$ (blue), and structurally relaxed singlet excited state $(S_1)_{STR}$ (red) obtained from GloTarAn target analysis. (d) – Relative population kinetics of the respective states.

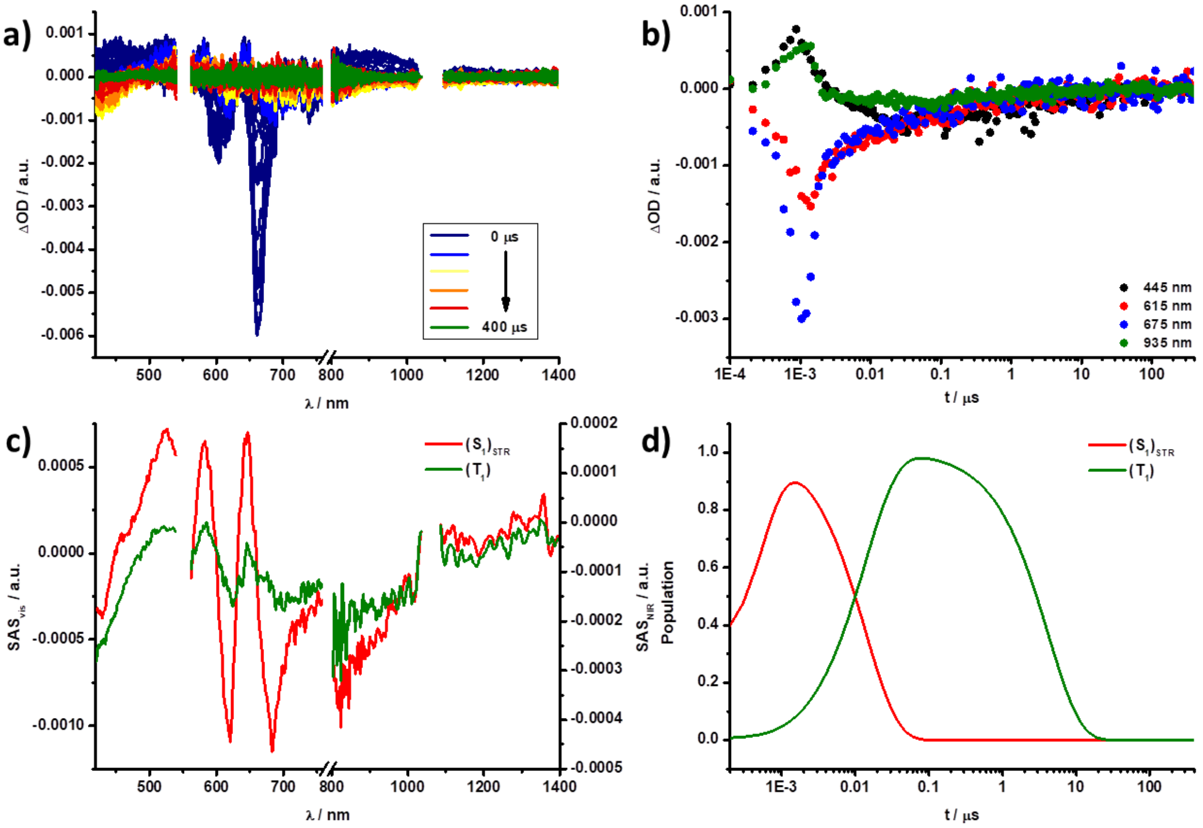

Figure S30. (a) – Differential absorption spectra obtained upon nsTA experiments (550 nm) of **6** in benzonitrile with several time delays between 0 and 400 µs at rt. (b) – Kinetics obtained from multi-wavelength analysis at the respective wavelengths. (c) – Species associated spectra of the transient absorption data of **6** shown in a), with the structurally relaxed singlet excited state $(S_1)_{STR}$ (red) and the triplet excited state (T_1) (green) obtained from GloTarAn target analysis. (d) – Relative population kinetics of the respective states.

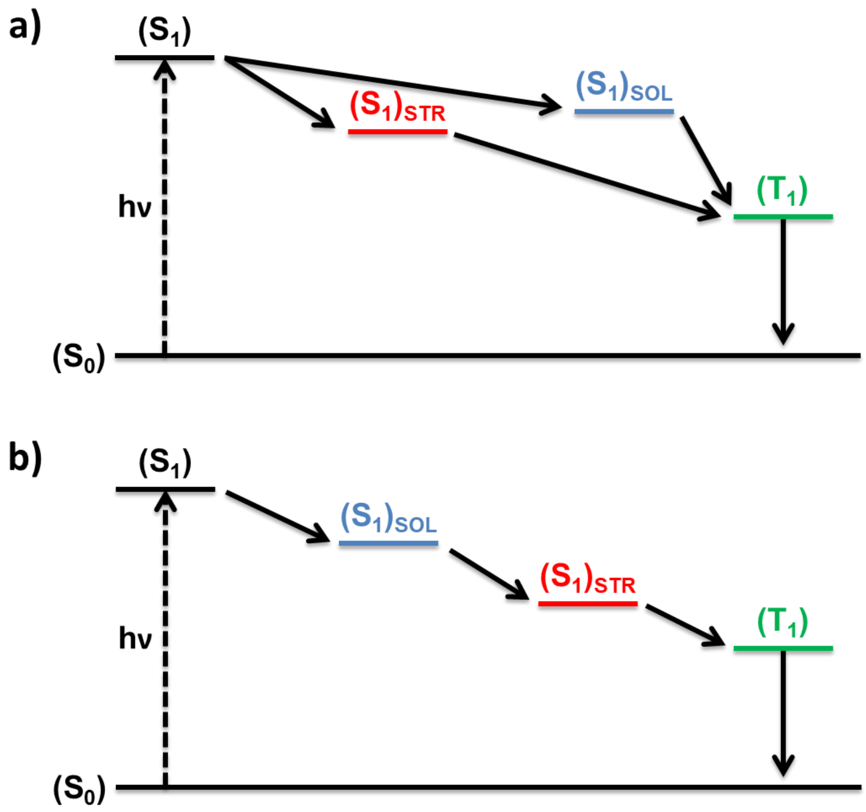

Figure S31. Applied kinetic model in the GloTarAn target analysis for fitting the transient absorption data of **3 – 6** in a) toluene and b) benzonitrile.

5. Computational Details

To model the steady-state optical spectra of the dyes, we have applied a hybrid protocol, in which the total and transition energies are determined with second-order Coupled-Cluster calculations (CC2), whereas the geometries, vibrations, and environmental effects are computed at the Time-Dependent Density Functional Theory (TD-DFT) level. All CC2 calculations were achieved with Turbomole[1] applying the resolution-of-identity approach and selecting the *aug*-cc-pVDZ atomic basis set. All (TD-)DFT calculations have been performed using the Gaussian16.A03 program.[2] For these calculations, we used tightened self-consistent field (10^{-10} a.u.) and geometry optimization (10^{-5} a.u.) convergence thresholds, and a large DFT integration grid (so-called *ultrafine* grid, a pruned 99,590 grid). These (TD-)DFT calculations relied on the M06-2X hybrid functional.[3] Following the basis set combination approach proposed in Ref. 4, we used the 6-31+G(d) atomic basis set for determining the geometrical and vibrational parameters (by default in gas, but some calculations have been made in solution when stated) whereas the transition energies have been computed with 6-311+G(2d,p) (in solution). The nature of the ground-state stationary points was confirmed by analytical Hessian calculations that returned 0 (minima) imaginary vibrational modes. Environmental effects (here, toluene and benzonitrile) on the transition energies have been accounted for using the polarizable continuum model (PCM).[5] For both absorption and emission we used a LR+cLR[6] model in its *non-equilibrium* limit, so as to capture both linear-response and state-specific solvent effects. Excited-states have been represented using density difference plots, in which the excited-state density was determined at the TD-DFT level. In these plots blue and red regions respectively indicate decrease and increase of electron density upon photon absorption. Vibrationally resolved spectra have been obtained using the FCclasses program.[7] The Franck-Condon (FC) approximation has been employed as we consider strongly dipole-allowed transitions (f>0.1), and we used the so-called Adiabatic Hessian (AH)[8] approach to determine the vibronic couplings. The reported spectra have been simulated by using convoluting Gaussian functions having a half width at half-maximum (HWHM) adjusted to reasonably reproduce the experimental broadness (ca. 0.06 eV). A maximum number of 25 overtones for each mode and 20 combination bands on each pair of modes were included in the calculation. The number of integrals to be computed for each class was set to at least 10^6, and it was tested that the FC factors exceeds 0.9 in all cases.

To investigate the nature of the excited-states under relaxation of the solvent and of the geometry, in connection with the transient measurements, it is crucial to correctly describe states of various natures, as the second state presents a strong CT character, and we have therefore turned to the ωB97X-D functional,[10] using, as above the 6-31+G(d) basis set for the geometry and frequency calculations that have performed with the PCM model applying both toluene and benzonitrile as solvents. Both the ground and the two lowest singlet excited, and the lowest triplet states were optimized in this way, using the LR-PCM approach in *equilibrium* was applied for the TD-DFT calculations (S_1 and S_2) and applying TDA-DFT with the same functional for the vertical triplet state (after ISC) and U-DFT for the relaxed triplet state. The total energies were next computed on these structures with the same functional, and the LR+cLR solvent model[6] applying both the *equilibrium* and *non-equilibrium* effects for the singlet excited-state, so as to estimate the solvent reorganization energy.

For the sake of computational effort, the side *t*-Bu groups substituting the benzofuran side groups were modelled as methyl groups.

(1) TURBOMOLE V6.2 2010, a development of University of Karlsruhe and Forschungszentrum Karlsruhe GmbH, 1989-2007, TURBOMOLE GmbH, since 2007; available from http://www.turbomole.com.

(2) M. J. Frisch, et al., Gaussian 16 Revision A.03, **2016**, Gaussian Inc. Wallingford CT.

(3) Y. Zhao and D. G. Truhlar, *Theor. Chem. Acc.*, **2008**, *120*, 215–241.

(4) B. Le Guennic. B and D. Jacquemin, D. *Acc. Chem. Res*. **2015**, *48*, 530–537.

(5) Tomasi, J.; Mennucci, B.; Cammi, R. *Chem. Rev*. **2005**, *105*, 2999–3094.

(6) Caricato, M.; Mennucci, B.; Tomasi, J.; Ingrosso, F.; Cammi, R.; Corni, S.; Scalmani, G. *J. Chem. Phys*. **2006**, *124*, 124520.

(7) F. Santoro, FCclasses, a Fortran 77 Code, 2011, code available at: http://village/pi.iccom.cnr.it

(8) F. Santoro, D. Jacquemin, *Wires Comput. Mol. Sci.* **2016**, *6*, 460–486.

(9) A. D. Laurent, C. Adamo, D. Jacquemin, *Phys. Chem. Chem. Phys.* **2014**, *16*, 14334–14356.

(10) J. D. Chai, M. Head-Gordon, *Phys. Chem. Chem. Phys.* **2008**, *10*, 6615–6620.

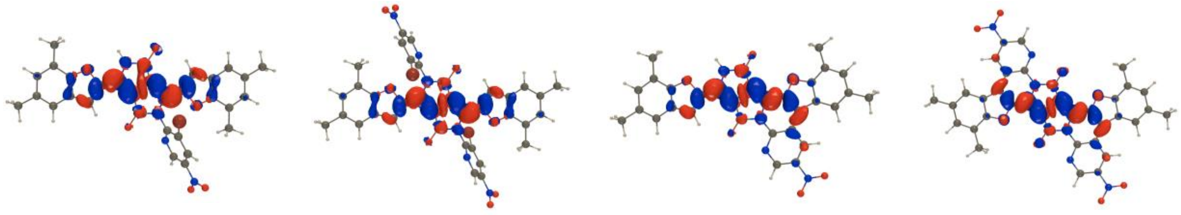

510 nm (*f*=1.225)	522 nm (*f*=1.288)	541 nm (*f*=1.132)	581 nm (*f*=1.216)
$\Delta\mu$ = +0.3 D	$\Delta\mu$ = 0.0 D	$\Delta\mu$ = -0.1 D	$\Delta\mu$ = 0.0 D

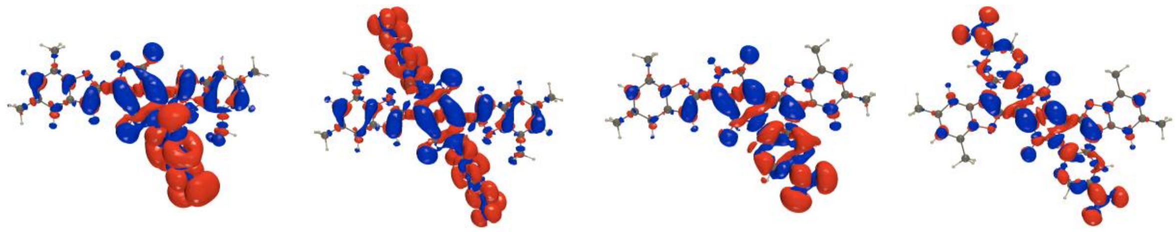

456 nm (*f*=0.010)	421 nm (*f*=0.000)	437 nm (*f*=0.102)	446 nm (*f*=0.000)
$\Delta\mu$ = +21.6 D	$\Delta\mu$ = 0.0 D	$\Delta\mu$ = +16.2 D	$\Delta\mu$ = 0.0 D
**3**	**4**	**5**	**6**

Figure S32: Representation of the density difference plots for the two lowest-lying states in the four synthesized compounds: top: S_0-S_1 transition, bottom S_0-S_2 transition as obtained at the FC point (S_0 geometry). The determined vertical transition wavelengths [CC2-corrected LR+cLR-PCM(Toluene)-TD-M06-2X values using toluene as solvent], are given as well together with the TD-DFT oscillator strength and the change of dipole moment upon transition. The contour threshold is 8 x 10^{-4} au.

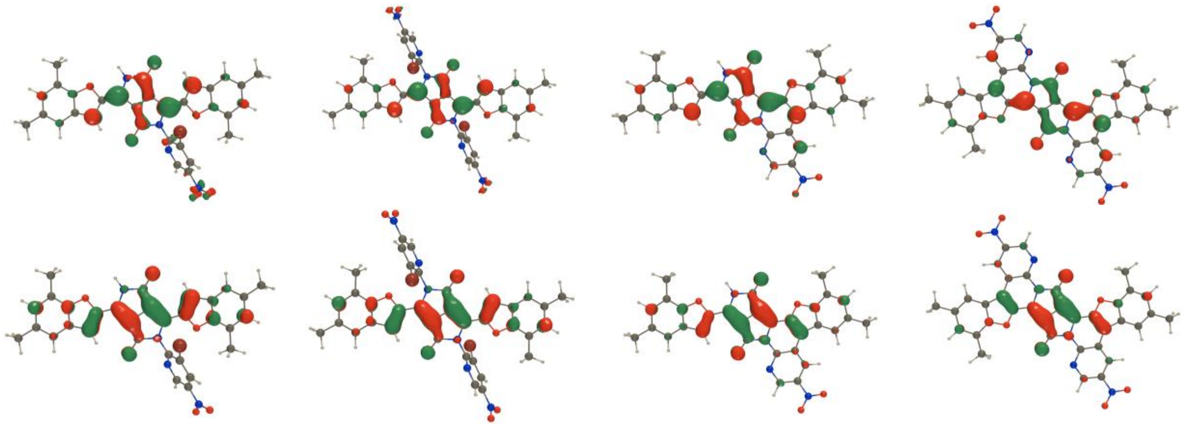

3 **4** **5** **6**

Figure S33. HOMO (bottom) and LUMO (top) of all compounds determined at the PCM(Toluene)-TD-M06-2X level of theory. Contour threshold: 0.04 au

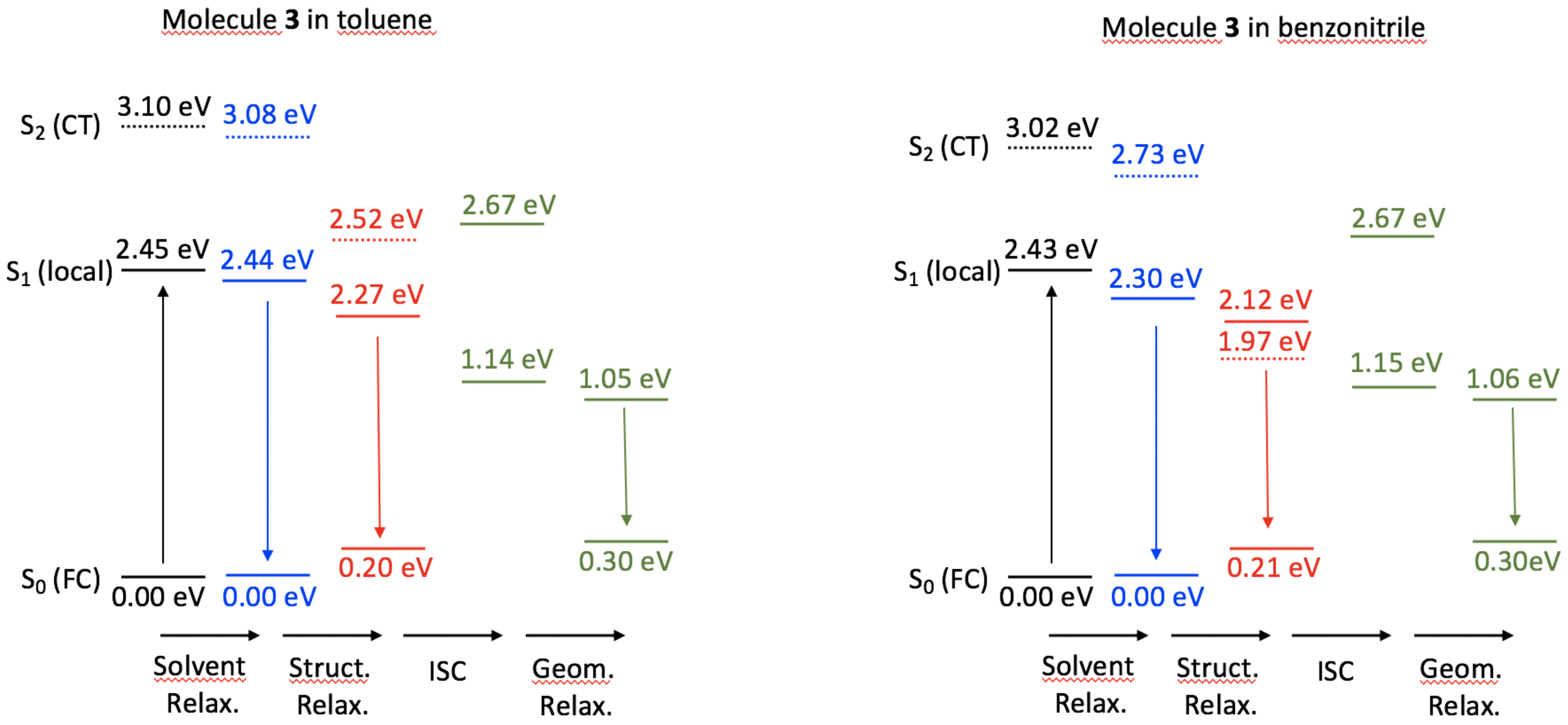

Figure S34. Relative ωB97X-D/6-311+G(2dp) energy levels determined for **3** in toluene and benzontrile taking the FC point as reference. The dashed lines correspond to the S_2 state.

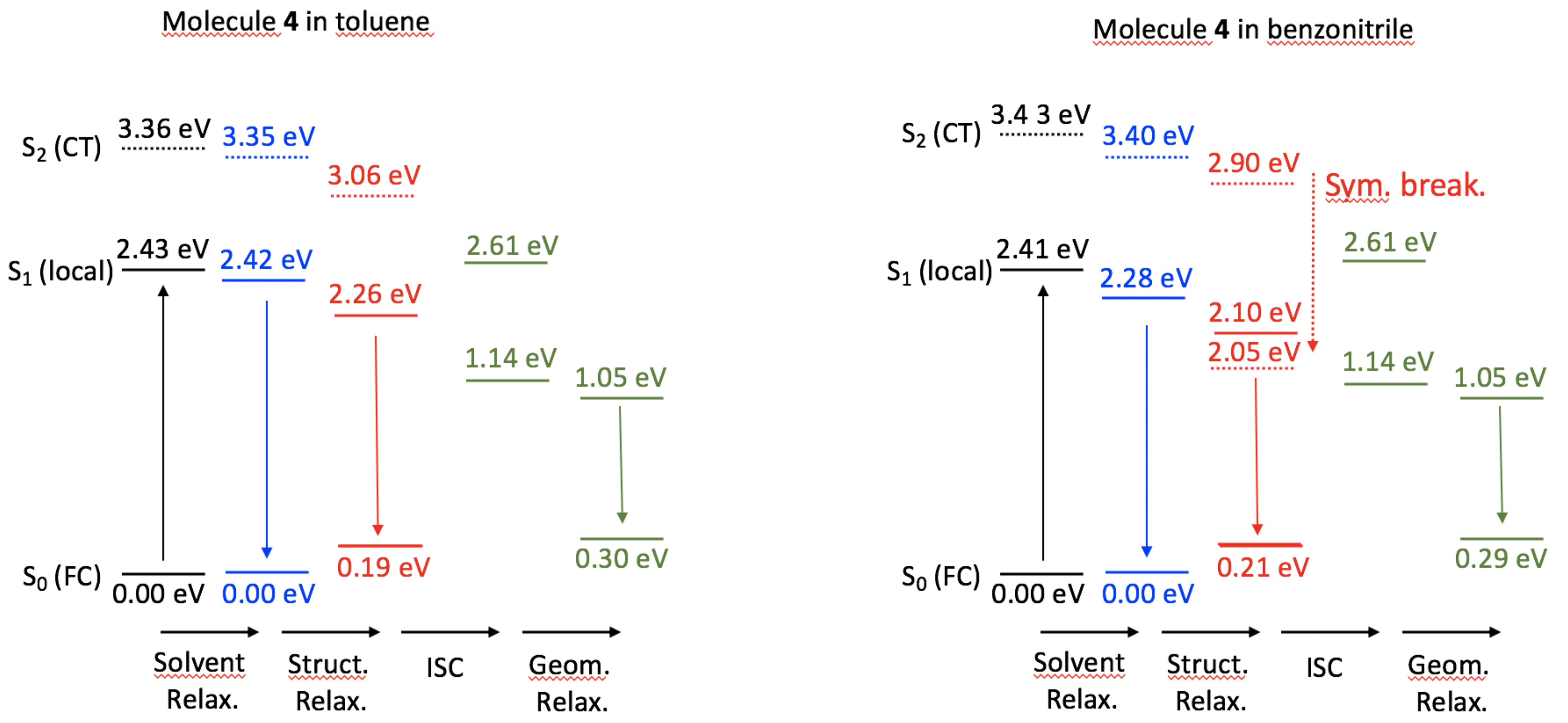

Figure S35. Relative ωB97X-D/6-311+G(2dp) energy levels determined for **4** in toluene and benzontrile taking the FC point as reference. The dashed lines correspond to the *S*$_2$ state.

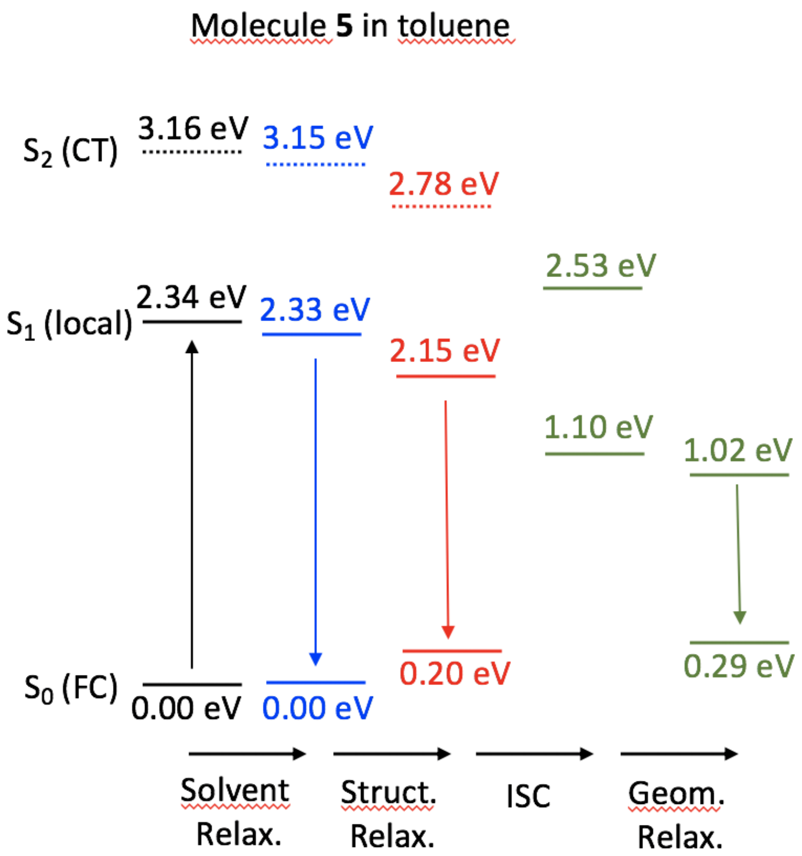

Figure S36. Relative ωB97X-D/6-311+G(2dp) energy levels determined for **5** in toluene and benzontrile taking the FC point as reference. The dashed lines correspond to the S_2 state.

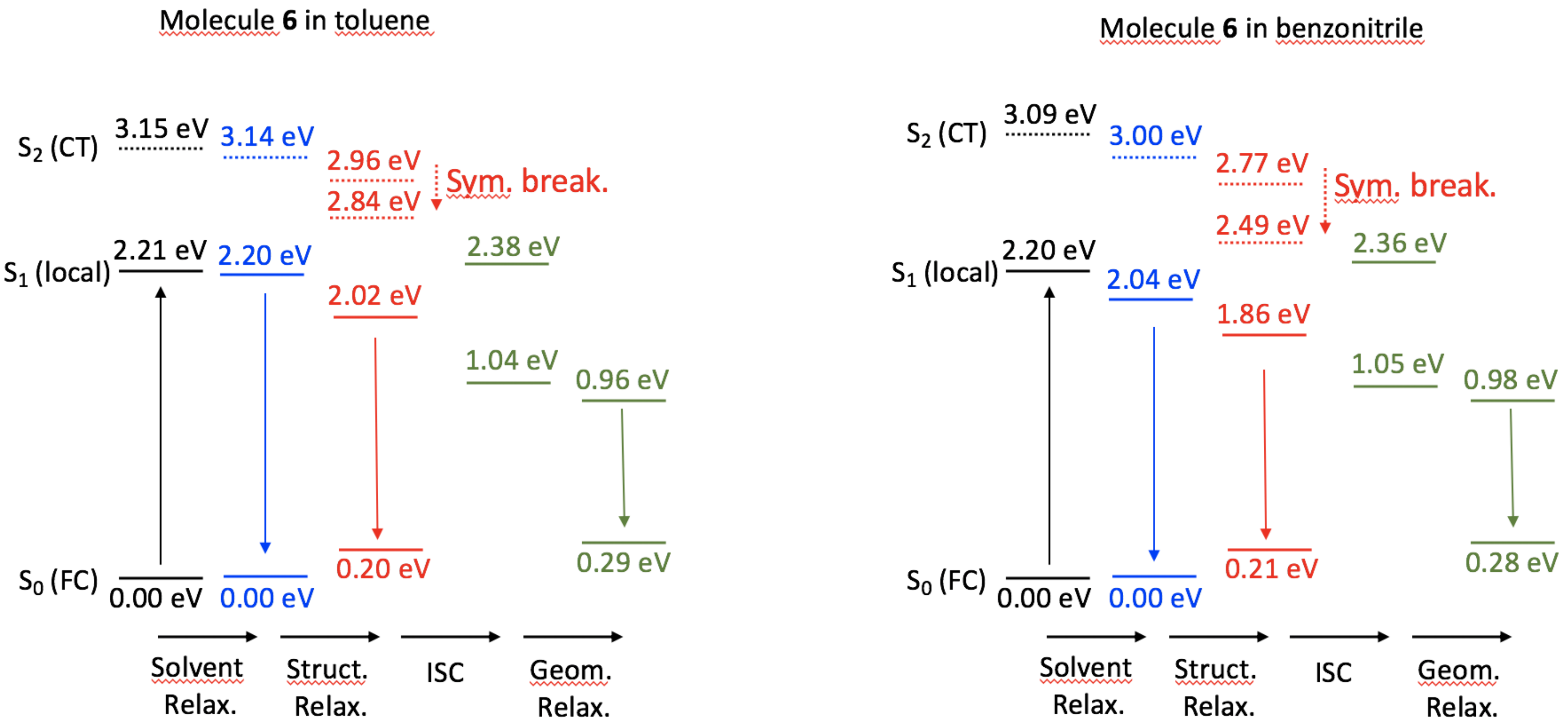

Figure S37. Relative ωB97X-D/6-311+G(2dp) energy levels determined for **6** in toluene and benzontrile taking the FC point as reference. The dashed lines correspond to the S_2 state.
